# Stromal vascular fraction in canine osteoarthritis: advantages, applications, and insights for veterinary practitioners

**DOI:** 10.3389/fvets.2025.1586629

**Published:** 2025-05-16

**Authors:** Aline Yen Ling Wang, Ana Elena Aviña, Yen-Yu Liu, Huang-Kai Kao

**Affiliations:** ^1^Center for Vascularized Composite Allotransplantation, Chang Gung Memorial Hospital, Taoyuan, Taiwan; ^2^International PhD Program in Medicine, College of Medicine, Taipei Medical University, Taipei, Taiwan; ^3^Department of Plastic and Reconstructive Surgery, Chang Gung Memorial Hospital, Taoyuan, Taiwan; ^4^College of Medicine, Chang Gung University, Taoyuan, Taiwan

**Keywords:** stromal vascular fraction, SVF, canine osteoarthritis, regenerative medicine, veterinary orthopedics, inflammation modulation

## Abstract

Canine osteoarthritis (OA) stands as a prevalent and excruciating joint condition that represents a promising application of stromal vascular fraction (SVF) treatment. In this review, we discuss the multi-factorial advantage of SVF cited as anti-inflammatory, regenerative, and angiogenic, all of which improve the complex pathology of OA. The heterogeneous cellular structure of SVF allows it to achieve joint improvement through both cell-based tissue restoration and signaling functions that benefit joint health. Veterinary practitioners need to consider essential aspects for clinical practice including patient selection criteria together with specific dosage recommendations along with additional therapies like platelet-rich plasma. Existing clinical data shows that SVF reduces pain while helping restore joint functions while practitioners face challenges when standardizing protocols and evaluating long-term safety aspects. Future research initiatives aim to translate advanced technologies including bioactive scaffolds, gene editing, and artificial intelligence which show promise for enhancing therapeutic results. This review integrates existing information about SVF while enlightening veterinarians about the gap areas to assist them make informed decisions when implementing SVF in practice. SVF represents a huge step forward in veterinary regenerative medicine because it enables better management of OA as well as other orthopedic applications.

## 1 Introduction

OA stands as the main chronic joint disease in dogs causing a severe decline in quality of life by introducing pain alongside stiffness and reduced mobility (1, 2). The condition of OA presents a serious problem in veterinary medicine since it targets 20% of dogs over 1 year old with even higher prevalence rates observed in older dogs (1, 3). The multiple causes of canine osteoarthritis include both joint instability disorders like hip or elbow dysplasia and cranial cruciate ligament rupture alongside excess weight-bearing that starts an inflammatory and biomechanical chain reaction in affected joints (3–5). Thus, a vicious cycle of joint degeneration is formed, with the main pathological mechanisms including cartilage degeneration, subchondral bone changes, synovitis, neuropathic and mechanical pain, joint instability and muscular atrophy (6–8). (1) Cartilage degeneration: As the sole cell type found in cartilage tissue chondrocytes manage both the formation of extracellular matrix (ECM) and its breakdown process. The dysregulation of chondrocytes in OA causes the production increases of catabolic enzymes including matrix metalloproteinases (MMPs) and aggrecanases which break down type II collagen along with aggrecan which forms the main cartilage structural components (9). The normal cartilage-building and cartilage-degrading processes become severely imbalanced thus resulting in weakened structural integrity of cartilage tissue. Cartilage has restricted self-healing capabilities because it lacks blood vessels and nerves which causes OA progression to become more acute. (2) Subchondral bone changes: The underlying bone stiffens and thickens as a response to cartilage loss. The disrupted shock-absorbing function of the joint becomes impossible as a result of this sclerosis which leads to pain accompanied by joint dysfunction (9). Bone spurs grow at the joint margins which serves as a distinctive feature of OA. Medical experts believe the development of osteophytes originates from joint instability yet these bone growths frequently lead to stiffness along with pain. Moreover, bone marrow lesions indicate microfractures and increased bone remodeling, worsening the disease. (3) Synovitis: The synovium develops inflammation which triggers excessive production of pro-inflammatory cytokines interleukin-1beta (IL-1β) and tumor necrosis factor-alpha (TNF-*α*) (9–13). The activity of MMPs intensifies because of cytokine stimulation which consequently speeds up cartilage damage rates. During inflammation, synovial macrophages additionally produce inflammatory mediators which worsen joint injuries. Moreover, altered synovial fluid composition like a decrease in hyaluronic acid content reduces the lubricating and shock-absorbing properties of the joint. (4) Neuropathic and mechanical pain: Joint movements produce heightened pain symptoms through central and peripheral sensitization processes which develop because of sustained inflammation (14, 15). Additionally, increased nociceptor activity within the joint contributes to chronic pain even in the absence of movement. (5) Joint instability and muscular atrophy: OA like ligament and meniscal Degeneration is often secondary to joint instability caused by conditions such as cranial cruciate ligament rupture, hip dysplasia, and elbow dysplasia. The prolonged experience of chronic pain causes animals to avoid using their limbs which subsequently leads to muscle atrophy and limb weakness that intensifies joint instability. Therefore, progressive degeneration of articular cartilage, subchondral bone sclerosis, osteophyte formation as well as variable degrees of synovitis are the hallmark of canine OA (1, 3, 5). The limited ability of cartilage regeneration becomes worse because cartilage lacks blood vessels and contains minimal chondrocyte activity (16). The chronic condition develops from joint cells undergoing unbalanced anabolic-catabolic activity which pro-inflammatory cytokines IL-1β and TNF-*α* frequently mediate (2, 3). Cartilage destruction and joint dysfunction exist within a continuous pathological cycle that involves these several factors.

Accurate osteochondral pathologies require a combination of clinical evaluation and imaging and biomarker analysis for proper diagnosis (17). (1) Clinical evaluation: The diagnosis of osteoarthritis requires gait analysis for observing weight-bearing and movement abnormalities by identifying limping alongside stiffness and rising difficulties (18). Joint swelling and pain along with crepitus are evaluated through palpation combined with a range of motion tests to measure mobility limitations. The Canine Brief Pain Inventory (CBPI) represents an objective pain assessment scale that aids standardized evaluation of both pain intensity and impact on the dog’s life quality (19, 20). (2) Imaging techniques: Radiographic imaging plays a crucial role in diagnosing osteoarthritis at different stages (21). The use of X-rays offers detection of advanced staging OA along with structural changes that include osteophytes, joint space narrowing, subchondral sclerosis, and bone remodeling though it lacks sensitivity for early cartilage damage identification (22). Magnetic Resonance Imaging (MRI) provides better imaging of soft tissues which enables doctors to identify cartilage degeneration and synovitis as well as bone marrow lesions at an early stage (23–26). Computed Tomography (CT) scans and ultrasound techniques assist in the detailed evaluation of joint structures by delivering information about structural bone shapes and tissue irregularities for patients with elbow or hip OA (27–31). (3) Biomarker analysis: The analysis of biomarkers serves as an important method to monitor osteoarthritis progression as well as determine its severity (29, 31–35). A synovial fluid analysis confirms joint inflammation when it shows elevated quantities of inflammatory mediators IL-1β and TNF-*α* and prostaglandins since these markers signal active inflammatory joint processes (36). Serum and urine biomarkers function as detectable indicators of cartilage degradation throughout the whole body (23, 28, 37). The measurement of cartilage oligomeric matrix protein (COMP) combined with C-terminal telopeptide of type II collagen (CTX-II) together with hyaluronic acid detection accurately demonstrates extracellular matrix breakdown thus supporting early disease detection along with condition assessment (38, 39).

Traditionally, symptomatically managing OA has been central to the OA therapeutic landscape rather than curing the problem, by decreasing pain, and inflammation, and improving mobility (3, 40, 41). The treatment of OA is based on pharmacological interventions with non-steroidal anti-inflammatory drugs (NSAIDs) such as carprofen, meloxicam and more recently coxib-class agents are the cornerstone of OA treatment since they are effective in controlling pain and inflammation (42–45). Nevertheless, these drugs have certain limitations such as gastrointestinal, renal, and hepatic side effects, especially in elderly patients (3, 42). The medical field faces a crucial unmet requirement due to the inability to stop disease advancement. Various complementary treatment options extending beyond medication use including weight control and physical therapy and nutraceutical supplement use with glucosamine and chondroitin sulfate have gained increasing popularity among patients (46–49). Symptomatic improvement can be obtained through these treatments yet scientists remain uncertain about their ability to restore completely damaged cartilage tissue. Extracorporeal shockwave therapy (ESWT) treatment and therapeutic ultrasound therapy show initial promise at both reducing pain and improving function according to research findings but available evidence lacks support for their sustained long-term benefits (50, 51). Joint replacement surgery alongside corrective osteotomies are surgical options for advanced arthritis but these procedures prove difficult and expensive to manage for elderly dogs (40). The rising number of dogs affected by OA has led researchers to develop regenerative medicine as an innovative solution due to the persistent restrictions of existing treatment methods.

Because canine OA is a progressive condition and joint tissues have limited regenerative capacity, canine OA is a significant clinical problem. The treatment methods using NSAIDs together with physiotherapy provide temporary symptom relief by not targeting the fundamental disease processes of OA (40, 42). Recent scientific studies show there is an urgent need to create new strategies that fight against symptoms and stimulate joint tissue restoration. In this case, adipose-derived stromal vascular fraction (SVF) has become a promising therapeutic possibility (52–59). SVF cells start from adipose tissue and contain mesenchymal stem cells (MSCs) and endothelial cells together with pericytes and immune modulatory components (60, 61). SVF activates tissue regenerative properties through its combined effect on inflammation control and cartilage restoration through paracrine and differentiation mechanisms (56, 59). Additionally, harvesting of SVF can be performed with scarcely cell death using mechanical or enzymatic processes that make it a feasible and available option for regenerative therapy in veterinary practice (52, 55). Clinical trials performed on canine subjects validated SVF as a potential treatment by enhancing lameness along with pain scores and joint functionality improvement in OA cases that did not respond to conventional medical approaches (54, 59). Single intra-articular SVF treatments have proven effective at decreasing both patients’ pain levels and their walking metrics according to studies (53, 56, 59). The regenerative treatments become more extensive when SVF works with other therapeutic techniques such as platelet-rich plasma and hyaluronic acid because these interventions generate observed synergistic effects. Nevertheless, while the concept promises, there are still obstacles to standardizing SVF isolation techniques, finding optimal cell doses and knowing the long-term effects of the SVF on joint health. The varying outputs from different studies regarding clinical results necessitate additional research that aims to establish reliable best practices while achieving reproducibility (52, 55, 57, 60). This paper reviews both the numerous benefits and specific implementations of SVF treatment in dogs with OA while exploring its associated difficulties. The review integrates existing evidence to provide veterinary practitioners with applicable lessons on SVF clinical usage strategies which examine both benefits and limitations. The presented discussion seeks to establish its significance in developing regenerative medicine practices for contemporary veterinary orthopedic care.

## 2 Biological characteristics of SVF

### 2.1 Composition and function

Adipose tissue-derived SVF functions as a heterogeneous group of cells that advances regenerative medicine because it supports multiple functions for tissue restoration and regrowth (60, 62, 63). SVF includes MSCs, endothelial cells, pericytes, preadipocytes, macrophages, and a variety of immune and progenitor cells (64–66). The primary advantage of SVF over expanded adipose-derived stem cells (ADSCs) is that SVF prepares at the “point-of-care” facility before direct therapeutic implementation without extensive processing steps (67, 68). SVF includes MSCs which represent its primary regenerative part because these multipotent cells can develop into chondrocytes and osteoblasts as well as into adipocytes (63, 69). The anti-inflammatory and immunomodulatory characteristics make these cells essential for cartilage repair. These include endothelial and progenitor cells, which are important for the promotion of angiogenesis, a process of restoring blood supply to the damaged tissues (64, 70). This increases microenvironment vascular support for the healing of the tissue. Blood vessel structures receive stabilizing support from pericytes that may display progenitor cell characteristics depending on specific circumstances (62, 65). Inflammation and immune responses are mediated by immune cells to remodel the tissue through regulating the balance of pro-and anti-inflammatory cytokines (63, 64). SVF acts through a number of mechanisms such as angiogenesis, immunomodulation, paracrine signaling, tissue remodeling, and regeneration (64, 65, 69). SVF stimulates the formation of new blood vessels that oxygen and nutrients the regenerating tissues need, through endothelial progenitor cells. Injected MSCs and macrophages in SVF also produce cytokines like interleukin-10, which dampens excessive inflammatory responses that may otherwise suppress healing. The release of growth factors such as vascular endothelial growth factor (VEGF) and transforming growth factor-beta (TGF-*β*) from SVF cells helps cell proliferation while also stimulating the development of extracellular matrix. MSCs and preadipocytes maintain multipotency which enables these cells to differentiate into chondrocytes directly while participating in a process that builds cartilage tissue. Adipose-derived SVF offers superior advantages over MSCs obtained from bone marrow including higher cell production amounts alongside minimally invasive processes and instant accessibility (62, 65). MSCs are seen to be present in adipose tissue at up to 500–1,000 times that number than in bone marrow. Bone marrow aspiration is more invasive than extraction of adipose tissue; therefore, patient discomfort is reduced for adipose harvest. The immunological processing of cultured ADSCs takes 1 week followed by 2 weeks of production but the SVF isolation process can yield immediate use which reduces regulatory limitations and expedites treatment schedules. SVF demonstrates utility in regenerative medicine because it contains multiple cell types that function well in complex medical situations like osteoarthritis. However, as the research continues, standardizing the culture methods and understanding the interplay between its cellular components will confer its therapeutic potential (67, 71).

### 2.2 Isolation and purification methods

The procedure for SVF extraction from adipose tissue needs specific step-wise controls to maximize cell yield together with cell viability and safety outcomes (62, 72). The regenerative potential of SVF makes it critical in veterinary medicine and medical therapies because its isolation remains easy and abundant while collection causes minimal invasiveness.

#### 2.2.1 Adipose tissue acquisition

Subcutaneous fat deposits or visceral fat constitute the main source of adipose tissue in animals (64, 73). These tissues can be harvested from peritoneal fat, omental fat, or subcutaneous regions when accessibility and cell yield are required according to demand, in canine applications. Many procedures are carried out under general anesthesia, and hence are ethical and safe. Collecting fat samples during routine surgeries, such as spaying, has the least amount of additional trauma. Immediately it is harvested the tissue is either processed directly for SVF extraction or preserved temporarily under sterile conditions. The choice of fat depot is significant as subcutaneous fat tends to yield higher MSC counts compared to visceral sources.

#### 2.2.2 SVF isolation methods

Isolation of SVF employs enzymatic or mechanical techniques, each offering distinct advantages and limitations (67, 68, 74). The extra cell matrix is digested effectively by collagenase, the enzyme of choice for enzymatic digestion, thereby releasing stromal and vascular cell components. This consists of specialized washing of the adipose tissue including the removal of impurities, enzymatic digestion at controlled temperatures, and subsequent neutralization. The method allows scientists to obtain large numbers of viable cells that contain heterogeneous populations including MSCs along with endothelial cells and pericytes. For example, we obtained 15–20 grams of subcutaneous adipose tissue in every SVF isolation procedure from the inguinal areas of canine subjects (75). The sterile phosphate-buffered saline (PBS) solution washed the tissue before researchers utilized 0.1% collagenase type I (Sigma-Aldrich, GMP-grade) under 37°C temperature for 30–45 min of agitation to achieve tissue digestion. The collagenase digestion solution also contained 10% fetal bovine serum (FBS; Wisent Inc.) and 1% antibiotic–antimycotic solution (Gibco-BRL). FBS addition helps to buffer enzymatic activity under collagenase digestion which prevents damage to stromal and stem cells while increasing the survival rate of mesenchymal stem cells. The antibiotic–antimycotic solution minimizes the risk of contamination during tissue processing. Gentle agitation was applied during the digestion to ensure even enzyme exposure and to facilitate efficient release of stromal and stem cells from the adipose matrix. A cell strainer with 100 μm pores filtered the digested mixture followed by 5 min of centrifugation at 1300 rpm to obtain the SVF pellet before saline-based resuspension for purification and injection procedures. Prior to resuspension, the SVF pellet was washed again with sterile PBS by centrifugation at 1300 rpm for 5 min to thoroughly remove residual collagenase, thereby minimizing potential enzymatic activity that could negatively affect the therapeutic application of the SVF solution. The entire procedure took approximately 90–120 min from harvesting to final SVF preparation. Stem cell viability suffered from increased enzymatic digestion times so scientists controlled the period carefully. The mechanical isolation technique separates cells through shear forces and centrifugation and filtration separation methods that work without added enzymes. Although it is faster and cheaper, the cell yield and viability may be less or vary than those obtained in enzymatic methods. However, mechanical procedures provide regulatory compliance and allow point-of-care usage for autologous methods in medical settings.

#### 2.2.3 Purification and concentration

Following isolation, the SVF suspension is purified for higher therapeutic potential (63, 76). SVF cells are separated from adipose tissue debris and remnants by a centrifugation step. So after the centrifugation, an SVF-rich pellet is resuspended in a physiologic buffer or medium depending on how it is going to be used subsequently. The filtration step removes the larger tissue fragments, resulting in a smoother cell suspension in order to minimize the chance of complications related to injection.

#### 2.2.4 Quality control and standardization

The therapeutic efficacy of SVF is entirely dependent upon quality control. These key steps involve cell viability testing, flow cytometry, and sterility testing (72, 77). The percentage of viable cells is critical for all clinical applications and it can be assessed by cell viability testing. Cell populations in the SVF can be characterized with MSCs (CD73^+^, CD90^+^, CD105^+^) present while hematopoietic markers (CD45^−^, CD34^−^) are absent (73). Ensuring that there is no microbial contamination during the processing is crucial for safety, and hence, important applicability in OA has been demonstrated for intraarticular use.

The standardization of SVF together with its therapeutic application necessitates proper measurement techniques for cell quantity assessment beyond viability and surface marker evaluation. Different approaches to cell counting exist based on experimental resources and the analysis needs of each study. Cell counting and differential analysis of SVF cells can be conducted using various methods depending on the laboratory’s resources (78). (1) When budgets are limited, manual cell counting through the use of hemocytometers together with Trypan Blue staining remains an established and dependable method. Researchers can easily identify non-viable (blue-stained) cells from viable (unstained) cells through dead cell-specific staining with Trypan Blue. The light microscope allows examination of cells mixed 1:1 with 0.4% Trypan Blue solution which reveals the SVF cell concentration in a hemocytometer through direct observation. The total number of viable cells in the counted grid is used to calculate the concentration using the following formula:


$$\begin{array}{l} \text{Cell concentration}\;(\text{cells}/\mathrm{mL})\\ =(\text{Number of viable cells counted}/\text{Number of squares counted})\\ \times \text{dilution factor}\times 10^{4} \end{array}$$

This factor (10^4^) accounts for the volume under each square of the hemocytometer (0.1 mm^3^ = 10^−4^ mL). Despite its affordable nature, this procedure demands significant time commitment along with human variable effects. (2) Laboratories operating on medium finances can increase efficiency by using automated cell counting systems. Current automated cell counters in the commercial market count total cells and estimate viability using image-based and impedance-based methods to produce quick results. Specific counting models mandate Trypan Blue staining yet other products function with fluorescence-based viability dyes or label-free systems methodologies. Components designed with automated systems decrease human experimental errors to produce uniformly consistent results. (3) The preferred method for analyzing SVF subpopulations and total cell measurements in high-end laboratories is flow cytometry. The evaluation of SVF composition through surface marker labeling shows its capability to analyze SVF contents using flow cytometry which identifies MSCs via CD90 and CD105 markers and endothelial cells by CD31 and hematopoietic cells through CD45 markers. The antibody-conjugated fluorochromes used for staining cells enable the laboratory to measure absolute counts in addition to relative proportions between cell types for clinical standardization and dose optimization purposes. Each analysis method starting with manual hemocytometry and progressing to automated counting and flow cytometry provides different advantages in line with existing infrastructure while working together for quality control and therapeutic standardization of SVF products.

#### 2.2.5 Storage and application

SVF becomes ready for direct medical procedures after its extraction such as joint injections or it can be cryopreserved for future clinical use (62, 72). The preservation methods typically include dimethyl sulfoxide (DMSO) as a cryoprotectant to preserve cell function while storing biological specimens. SVF cells achieve long-term preservation through liquid nitrogen treatment which stops all metabolic functions and biochemical operations at its ultra-cold temperature point of −196°C. The cells transition into a vitrified state while cooled to −196°C because this temperature solidifies water on both cellular and extracellular levels without creating harmful ice crystals which ensures cell structural alignment with viability maintained. A proper preservation process for SVF cells involves initial storage at −80°C for pre-cooling before depositing them into liquid nitrogen containers. The storage conditions at liquid nitrogen surpass those available in −80°C freezers because residual metabolic activity and ice crystal formation do not occur making it ideal for long-term preservation. Cell viability together with functional capacity remains high for several decades when SVF cells are properly preserved under conditions maintaining suitable nitrogen levels.

#### 2.2.6 Innovations and standardization

The focus of current research has led to automated devices for SVF extraction that improve consistency and protect transparency in the procedure (67, 68, 74). They streamline the system to create a closed system environment where the risk of contamination is decreased. SVF makes it the essential foundation of regenerative medicine applications due to its established isolation and purification methods. Optimization of techniques and rigorous quality control of SVF enhances its clinical utility including its use to treat osteoarthritis and other conditions. Progressive innovations are made to optimize these protocols for the best therapeutic outcomes while being secure and available for human and veterinary applications.

### 2.3 Mechanisms of action

**Figure 1:**
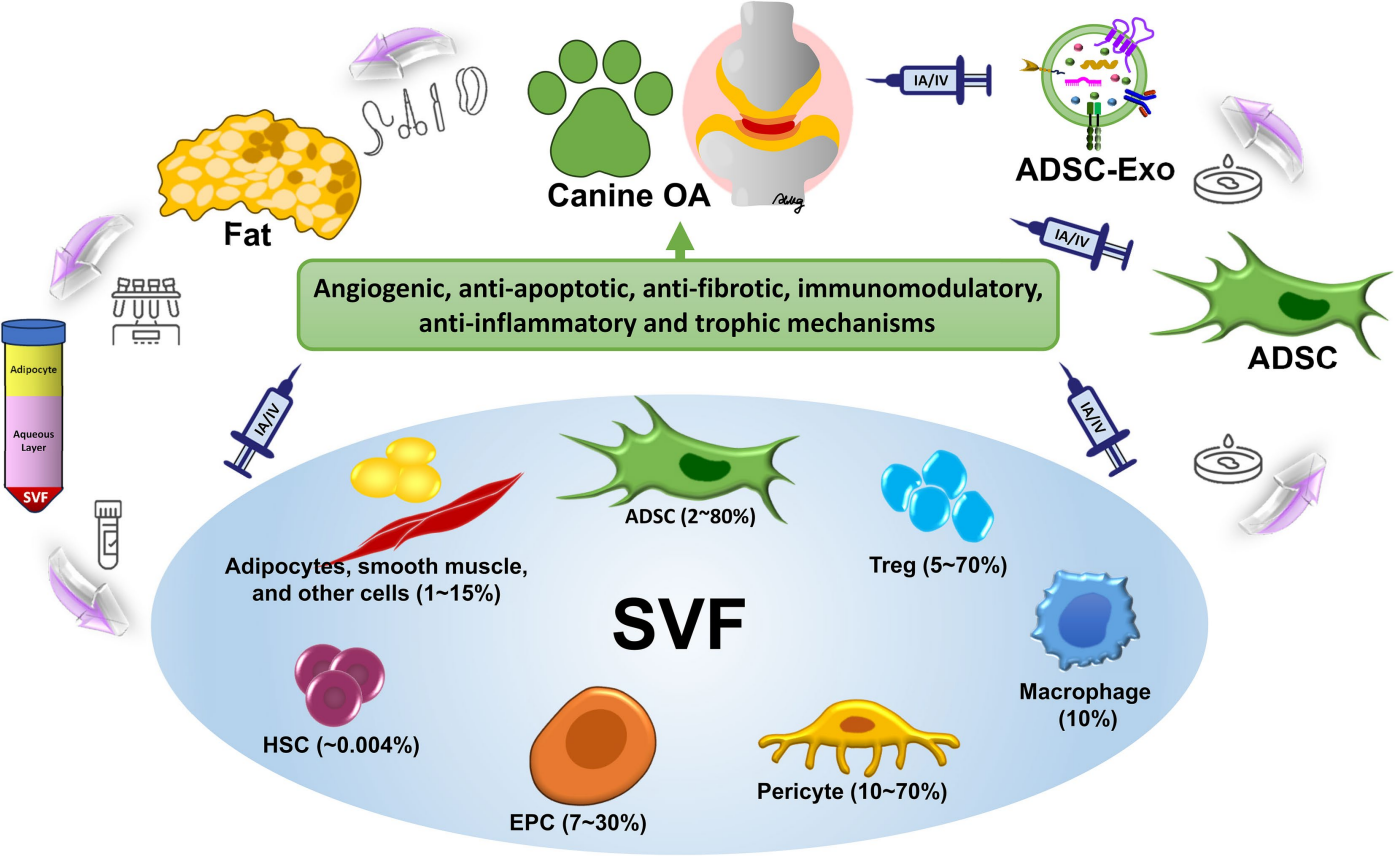
Mechanisms and delivery routes of SVF therapy in canine osteoarthritis. The illustration is the complete process and therapeutic use of SVF therapy in canine OA. SVF isolation from adipose tissue consists of enzymatic digestion and centrifugation to obtain regenerative cells such as ADSCs, endothelial progenitor cells (EPC), macrophages, regulatory T cells (Treg), hematopoietic stem cells (HSC), etc. Therapeutic use is then prepared for these cells. SVF therapy functions through three primary mechanisms which increase anti-inflammatory activities (pro-inflammatory cytokines like TNF-*α* and IL-1β reduction while elevating anti-inflammatory cytokines like IL-10 release) and also support angiogenesis through endothelial progenitor cell involvement and further stimulate cartilage regeneration by facilitating ADSC-mediated extracellular matrix synthesis and repair. The intra-articular (IA) delivery method targets joint tissue affected by inflammation and degeneration as a way to administer the treatment while the intravenous (IV) method delivers the treatment through blood circulation to provide systemic anti-inflammatory benefits for multiple joints. This integrated study demonstrates that SVF therapy possesses effective capabilities for symptom reduction and joint healing which leads to enhanced joint mobility in dogs affected with OA.

Multiple cellular mechanisms found within the SVF as a heterogeneous cell population derived from adipose tissue enable critical treatment of OA. Immunomodulation, promotion of angiogenesis, anti-inflammatory activity, as well as, facilitation of regeneration of tissue are these mechanisms (63, 64, 66, 79, 80). Together, these pathways suggest that SVF is a promising candidate for regenerative therapy of OA. As shown in Figure 1, SVF therapy in canine osteoarthritis involves mechanisms and delivery routes that could provide an opportunity as a regenerative therapy through immunomodulation, angiogenesis, anti-inflammatory activity, and tissue regeneration activities.

#### 2.3.1 Immunomodulation

Most importantly, SVF cells, and specifically MSCs along with macrophages, modulate immune response (62, 73). The MSCs of SVF are able to reduce the production of pro-inflammatory cytokines including TNF-*α* and IL-1β, and increase concentrations of anti-inflammatory cytokines such as interleukin-10 (IL-10). The change toward anti-inflammatory properties minimizes joint inflammation which is characteristic of OA. Moreover, MSCs interact with T cells to inhibit their proliferation and activation as well as to modulate the immune landscape. Macrophages in SVF also contribute significantly to immunomodulation. Macrophages in OA tissue develop from pro-inflammatory M1 to anti-inflammatory M2 polarization inside the microenvironment which allows tissue repair by creating a repair-permitting environment (81).

#### 2.3.2 Promotion of angiogenesis

The endothelial progenitor cells (EPCs) among SVF cells alongside VEGF secreted by MSCs drive angiogenesis (65, 69). EPCs help the formation of new blood vessels, a process called angiogenesis, which supplies oxygen and nutrients to damaged tissues. The secretions from SVF cells include VEGF along with other angiogenic factors that drive endothelial cells to proliferate and migrate thus developing blood vessels specifically in subchondral bone tissue and synovial membrane of OA joints. Through angiogenesis tissue metabolism receives support while the vascular supply of cartilage and subchondral bone reestablishes its integrity for maintaining joint homeostasis.

#### 2.3.3 Anti-inflammatory effects

The cellular components in SVF directly fight inflammation through their release of TGF-*β* and prostaglandin E2 (PGE2) molecules and demonstrate antioxidant effects (63, 77). The effects of these compounds are to inhibit the inflammatory pathways stimulated by nuclear factor kappa B (NF-κB) and therefore diminish inflammatory cell recruitment and cytokine release. SVF cells achieve their pain relief and reduced stiffness effects through their paracrine activity that regulates synovial fibroblasts and immune cell actions within the joint space.

#### 2.3.4 Tissue regeneration

SVF therapy achieves its main function through tissue regeneration of cartilage along with other joint tissues according to studies (63, 69). The MSCs of SVF possess the ability to differentiate into chondrocytes which make up cartilage and conduct both cartilage generation and upkeep activities. In addition, these MSCs make proteins of the ECM, such as collagen and proteoglycans, which are critical to cartilage integrity. SVF enhances its ability to remodel the matrix through its production of matrix metalloproteinase inhibitors that stop cartilage destruction. SVF cells promote cartilage repair partly through their release of growth factors that includes insulin-like growth factor-1 (IGF-1) and fibroblast growth factor (FGF).

#### 2.3.5 Paracrine signaling

Most therapeutic actions of SVF happen through sending specific signals to other cells using paracrine mechanisms (62, 65). The cells release cytokines together with growth factors and exosomes which enable cell-to-cell communication to enhance regeneration while reducing inflammation. Paracrine signals are essential in the signaling of the intricate cell interaction necessary for joint repair. The extensive therapeutic properties of SFV for OA treatment include immunomodulation as well as angiogenesis and inflammation reduction which together lead to tissue regeneration. The healing actions through these methods focus on both managing symptoms while directly addressing the root causes which aid complete joint restoration. The clinical value of SVF in veterinary practice and medical medicine will improve with ongoing research that optimizes its application methods and investigates its sustained effects.

## 3 Clinical applications of SVF in canine osteoarthritis

### 3.1 Current clinical research

Studies have increased appreciation for SVF therapy as an investigational drug for managing dog OA which often causes persistent pain while reducing their mobility. Studies on SVF safety as well as its effectiveness and extended therapeutic benefits have been carried out in clinical medical trials. Therefore, a systematic review of major research conducted on canine osteoarthritis enabled us to understand SVF’s effectiveness for this condition. The studies presented in Table 1 underwent thorough rigorous selection. Using the PubMed database, a total of 18 papers were identified using the keywords “stromal vascular fraction, canine osteoarthritis” in their titles and abstracts. An additional step of screening proceeded to narrow the selection process. The two authors independently assessed and double-confirmed each paper to determine studies dedicated to SVF effectiveness in treating canine osteoarthritis. Seven studies specifically examined the effectiveness of the SVF for treating osteoarthritis in canines. These studies form the foundation for the data presented in Table 1. A 2024 prospective research involved 23 dogs with advanced elbow OA who received a single intra-articular autologous SVF injection (59). The study documented substantial progress in lameness reduction together with pain score and gait analysis measurement improvements through the three-month evaluation period as 33% of cases achieved measurable outcomes. The OA condition stayed stable in 19 out of the 23 affected joints treated with the procedure. Research data demonstrates that SVF injections lead to symptom decline in dogs without noticeable adverse results. Where hip OA analysis involved dogs in 2016 researchers determined SVF alongside platelet-rich plasma (PRP) techniques produced major CBPI scoring and Peak Vertical Force (PVF) force improvement through a 24-week treatment period (54). The combination therapy in this study led dogs to have better limb functionality than placebo groups. Results from individual dog patients exhibited variations but the specific disease factors that influence treatments might differ between patients. A pilot study on elbow OA treated 19 dogs with SVF and PRP (82).

**Table 1:** Summary of clinical studies on SVF for canine joint disorders, including osteoarthritis and related conditions.

Disease	Sample size	SVF dosage	Treatment	Outcome measure	Result	Adverse event	Limitation	Reference
Osteoarthritis	Dogs (*n* = 22): SVF + PRP (10), placebo (12)	170 × 10^6^ cells per joint	Intra-articular and IV SVF + PRP	Lameness score, CBPI, VAS, PVF, VI, and radiographic analysis	Improved CBPI scores and PVF in treated group at 24 weeks; no radiographic differences	SVF + PRP administration showed no adverse reactions; a few cases exhibited transient mild lameness following the injection, which resolved without intervention	Small sample size; no long-term follow-up beyond 24 weeks; varying disease severity	Upchurch et al. (54)
Osteoarthritis	19 dogs with elbow OA	178 × 10^6^ cells per joint	Single intra-articular SVF + PRP	Lameness scoring, kinetic/kinematic gait analysis, radiographic assessment, and owner-reported outcomes	Improved lameness scores at 6 months; fore-hind symmetry improved at 12 months	Two dogs exhibited mild short-term adverse reactions, including localized swelling and transient pain.	Small sample size; no significant change in objective variables; no placebo group	Bergström et al. (82)
Osteochondral injury	Dogs (*n* = 12): PRP (4), SVF (4), SVF + PLGA (4)	N/M	Intra-articular SVF or SVF + PLGA	Functional, radiographic, biochemical, and histological assessments	PRP improved lameness and function at 6 months, while SVF showed no significant benefits, and SVF with PLGA worsened outcomes, raising safety concerns.	The combination of SVF with the PLGA scaffold resulted in adverse effects, suggesting potential incompatibility or harmful interactions during intra-articular injection, raising concerns about its safety and efficacy	Variability in SVF preparation, dosing protocols, small sample sizes, and limited objective assessments led to inconsistent results, while advanced OA stages and safety concerns with injectable scaffolds like PLGA further challenged efficacy	Franklin et al. (83)
Degenerative joint disease	10 dogs with DJD	I.V. 2 × 10^6^ allogenic cells/kg	Intravenous allogenic SVF	Radiographic assessment, pain score (CMPS-SF), VEGF levels, and owner-reported mobility	Significant reduction in pain within 1 week; improved mobility sustained for 6 months	No adverse reactions or complications were reported during or after the treatment	Small sample size; lack of placebo group; no long-term imaging results beyond 6 months	Kemilew et al. (58)
Osteoarthritis	23 dogs with elbow OA	8.2 × 10^6^ cells per joint	Single intra-articular SVF injection	Lameness scoring, gait analysis (PVF, VI), owner questionnaires, and radiographic evaluations	Significant improvement in PVF at 3 months and VI at 6 months; minimal radiographic changes	The treatment showed no significant side effects or complications	Lack of blinding; subjective lameness scores affected by potential placebo effect	Bruns et al. (59)
Hip dysplasia	Dogs with HD (*n* = 9): autologous SVF (4), allogeneic ADSC (5)	2–5 × 10^6^ cells per joint	Acupoint intra-articular injection	Lameness scoring, gait analysis, owner satisfaction	Improved mobility and lameness scores; reduced pain at 30 days	Both treatments were well-tolerated without significant side effects, and acupoint injections caused no discomfort to the animals	Small sample size; no placebo or control group; lack of long-term follow-up	Marx et al. (85)
Osteoarthritis	9 dogs with advanced OA	10.2 × 10^6^ cells per joint	Intra-articular autologous SVF	Lameness scores, gait analysis (PVF, VI), CBPI, HVAS, range of motion, radiographic analysis	Limited or short-term improvement in lameness; no significant long-term benefits	Except for one dog that required additional pain medication after treatment, no significant side effects were observed	Heterogeneous disease history and morphometry; small sample size; short follow-up	Schroers et al. (56)

N/M, Not Mentioned; SVF, Stromal Vascular Fraction; PRP, Platelet-rich Plasma; CBPI, Canine Brief Pain Inventory; VAS, Visual Analog Scale; PVF, Peak Vertical Force; VI, Vertical Impulse; OA, Osteoarthritis; PLGA, poly(L-lactide-co-glycolide); DJD, Degenerative Joint Disease; VEGF, Vascular Endothelial Growth Factor; I.V., Intravenous; ADSC, Adipose-derived Stem Cells; HD, Hip Dysplasia; HVAS, Hudson Visual Analogue Scale.

The combination of improved gait symmetry and clinical lameness scores occurred during a six-month treatment period yet these results failed to continue at the 12-month follow-up period. The owners’ assessment of improvement did not match the range of joint motion tests and X-ray results showing that certain evaluation methods are subjective and require standardized assessment protocols. The comparison between SVF therapy and PRP and SVF with poly(L-lactide-co-glycolide; PLGA) scaffolds for osteochondral injuries appeared in a 2018 research paper (83). Researchers found optimal results in terms of cartilage health markers and lameness assessment within the PRP treatment group. Utilizing SVF treatment alone produced moderate therapeutic outcomes but actual treatment results worsened when scaffolds were introduced leading to increased safety concerns about scaffold implementation. Research on SVF versus cultured MSCs demonstrates that SVF collects simpler while ready for application right away but cultured MSCs provide better potential for reparation through concentrated cell quantities and refined cell populations (84, 85). In spite of its constraints regarding *in vitro* growth regulation restrictions SVF proves more suitable for veterinary medical needs. Often one sees an improvement in lameness and joint mobility weeks to months following SVF treatment (56). However, research indicates that these effects tend to fade away over time specifically for dogs with severe osteoarthritis experiencing minimal long-term benefits at the six-month mark. It still remains a challenge. The outcomes from SVF therapy depend on baseline OA severity along with the injection method and the use of PRP treatment and different extraction techniques used to create SVF. SVF therapy administration shows good tolerance outcomes because patients experience only minor adverse effects involving localized swelling and brief pain following intravenous or intra-articular injection (54, 56, 58, 59, 82–85). Safety has been supported by no reported cases of systemic immune reaction or severe adverse events. Cultured or allogenic stem cells, however, have multiple ethical and regulatory issues that may be avoided by this approach to SVF using an autologous source from the patient’s own adipose tissue. Because of this, it is an accessible option for clinical application in dogs. The following are what most studies use to check the SVF efficacy (54, 56, 58, 59, 82–85). As an example, owner-reported outcomes (lameness and quality of life scores such as CBPI), objective measures (gait analysis, peak vertical force and symmetry indices, radiographic evaluation of joint degeneration), clinical examination scores (lameness scoring, pain on palpation, range of motion). The studies involve < 30 dogs, and the generalizability of findings is limited (54, 56, 58, 59, 82–85). Most trials are short-term (3–6 months) and thus there is a gap regarding sustained efficacy over the years. The analysis of multiple research studies becomes difficult due to inconsistent methods used in separating SVF and differing dosage approaches. The safety of SVF therapy makes it an appealing and safer treatment option for dogs with OA, particularly for those who failed to respond to NSAID and surgical treatments. The short-term benefits of SVF therapy in clinical trials do exist but both its lasting effects and how patients consistently respond to treatment require additional research study. Improved research methods and standardization of protocols together with combination therapy such as SVF with PRP may advance outcome results toward making SVF an established component of canine OA management systems.

### 3.2 Dosage and administration routes

SVF’s clinical use for treating canine OA depends significantly on the established dosing and administration procedures. There have been studies focusing on intra-articular and intra-venous injections with slightly varying outcomes on clinical outcomes (54, 56, 58, 82, 85).

#### 3.2.1 Intra-articular injection

The most established means of administration of SVF are via IA injections, providing high SVF regenerative cell concentrations directly into the joint space (54, 56, 82, 85). Such localized application allows SVF to have anti-inflammatory effects, cartilage repair effects, as well as systemic and tissue-level modulation of the diseased joint microenvironment. The delivery of medication into the joint through IA injection limits how much enters the system while it maintains higher concentrations within the treatment area. Usual dosages are injected 1–2 mL of SVF suspension with about 10–20 million viable cells into each joint. The quantity is often derived from processing 10–20 grams of adipose tissue harvested from the patient. Current research employs single doses for treatments yet additional examinations demonstrate that regular injections every 6 to 12 months could sustain relief in persistent cases. Research reveals substantial improvement in pain symptoms together with better gait symmetry and owner-perceived pain reduction after 3–6 months post-injection therapy. These effects may fade after a year and, therefore, these may require booster injections later. The use of IA injections promotes minimal disease progression while also demonstrating some joint space improvement with reductions in osteophyte formation. The treatment produces minimal adverse effects which primarily consist of passing joint discomfort and swelling. No infections or system immune reactions have been documented to be adverse.

#### 3.2.2 Intravenous injection

Systemic distribution across multiple joints or in the treatment of systemic factors related to OA is utilized via IV administration (58). The biological property of MSCs within the SVF involves their ability to travel and reach areas of inflammation such as joints for factor production that reduces inflammatory responses while supporting healing processes. Cells are given IV and typically range from 1 to 5 million cells per kilogram of body weight. For infusion, SVF is prepared in saline or a similar medium. Other studies give single doses or deliver weekly infusions over periods of 4–6 weeks in order to achieve cumulative benefits. IV administration has promise for the lowering of systemic inflammatory markers such as TNF-*α* and IFN-*γ*. Research studies find that patients obtain better mobility and pain management outcomes during therapy periods that involve IA injections alongside systemic administration of IV. Systemic administration of IV displays excellent promise for systemic treatment but monotherapy lacks the ability to deliver adequate localized benefit for fighting progressed joint damage therefore it works best as an auxiliary treatment with other approaches. Theoretical risks together with pulmonary microembolism present low potential in dogs since no severe negative events have been documented from the study. The infusion rate needs precise adjustment alongside dose calculation in order to limit possible adverse outcomes.

#### 3.2.3 Combination therapy

The combination of IA and IV administration is to attempt optimized local and systemic effect. Single route administration has resulted in inferior outcomes, having demonstrated worse lameness and joint function when compared to the present approach (54, 83). SVF receives enhanced effects when combined with PRP due to the additional growth factors such as TGF-*β* and VEGF present in PRP that facilitate cartilage repair and show anti-inflammatory properties. These studies have shown that IA co-administration of SVF and PRP provides synergistic benefits including, greater pain relief and maintained function (54). When administered in combination, IA and IV SVF appear to result in enhanced outcomes compared to that of single modality approaches, without further augmentation of the safety concerns associated with either IA or IV SVF treatment alone.

Research comparability struggles because scientists follow different approaches when preparing cells, setting doses, and measuring viability (52, 55, 57, 60). All research must adopt universal standardized protocols (60, 67, 72). The initial positive research findings need additional studies about booster therapies and supplementary treatment methods to achieve consistent sustained results (54, 56, 59, 82–85). A new generation of closed-system devices designed for SVF preparation at the point of care would enhance both the preparation process and the consistency of its results (67, 68, 74). Further improvements in large therapeutic effects can be possible by combining SVF with anti-inflammatory drugs, hyaluronic acid, or scaffold-based delivery systems (70, 83, 86). As a result, intra-articularly injecting SVF remains the gold standard technique for delivery of SVF in canine OA due to localized direct effects and minimal risks (54, 56, 59, 82, 85). Although their administration systemically, it is best used adjunctively intravenously (54, 56, 59, 82, 85). Upcoming combination approaches—especially those incorporating PRP—have shown some promise in the ‘maximization’ of therapeutic outcomes (54, 82, 87). Much more work is needed in further large-scale, controlled studies to refine protocols, improve long-term efficacy, and establish SVF as a cornerstone therapy for canine OA (59, 82, 85).

### 3.3 Clinical efficacy assessment

Clinical, biomechanical, subjective, and imaging-based measures are combined for the assessment of the therapeutic efficacy of SVF in canine OA (54, 56, 59, 82). The goal of these methods is to assess improvements in joint function, pain relief, and structural integrity, thus providing a more complete picture of treatment outcomes.

#### 3.3.1 Lameness scoring

Lameness scoring is a commonly used assessment that semi-quantifies limb function. A 0–4 grading scale is typically used by veterinarians. Grade 0 indicates no observable lameness. Grade 1 lameness is detected only during certain activities and is very subtle. The severity of OA worsens from Grade 2 through Grade 4 according to the degrees of pain that occur when dogs walk or rest weight on their elbows. Investigators have revealed that SVF therapy administered into the joint space of elbow OA patients effectively reduced lameness scores during a 2024 clinical study that included 23 participants (59). The evaluation of 19 dogs that received SVF in combination with PRP treatment showed a lameness score improvement of 50% at the 12-month follow-up however disease severity impacted the outcome (82).

#### 3.3.2 Gait analysis

A biomechanical evaluation of OA treatments depends heavily on objective gait analysis. Force plates and pressure-sensitive walkways enable researchers to collect data that includes PVF and Vertical Impulse (VI). The weight-bearing capacity of affected limbs has a direct correlation to the values obtained during assessment which provides detailed information regarding functional recovery. A functional improvement of PVF values occurred in 43% of dogs 3 months after receiving an SVF injection. The research team analyzed VI measurements at 6 months to prove that functional gains lasted (59). Bilateral OA cases presented significant improvements based on symmetry index measurements that checked fore-hind leg weight distribution. These improvements show variabilities in maintaining them for more than 12 months (long-term studies) for which booster doses or adjunct therapies have been necessary (59).

#### 3.3.3 Owner-reported outcomes

Important information about the quality of life changes after treatments comes from the subjective evaluations reported by owners of the dogs. The measurements of animal pain and lameness levels can be assessed through CBPI together with Hudson Visual Analogue Scale (HVAS) which are administered by owners. In a 2024 study, CBPI proved it reduced pain significantly in more than 26 percent of dogs over 6 months, as reported by owners (59). The owners monitored pain relief in 67% of dogs that maintained their pain-improvement state during the 12-month follow-up period. The owners documented both clinical observations and their pet’s performance of everyday activities like stair climbing and playful behavior.

#### 3.3.4 Imaging and structural evaluations

Radiographic imaging and magnetic MRI are employed to evaluate the structural impacts of SVF therapy. The focus of these methods is on various parameters of the image such as joint space narrowing, osteophyte formation, and cartilage integrity. Radiographic evidence showed minimal joint progression in 19 out of 23 elbows treated with SVF according to the 2024 elbow OA study (59). The research provides evidence that SVF protects joints from developing structural damage. In cases where SVF has been combined with PRP, MRI showed improved cartilage thickness and decreased inflammatory synovial fluid volume in some dogs, in conjunction with clinical improvement. As valuable as imaging is, less change occurs in the later (advanced) stages of OA. Such imaging biomarkers could be useful as targets for future studies of early detection of therapeutic effects.

#### 3.3.5 Advanced biomarkers

A new approach to evaluating drug effectiveness through biomarkers has started to appear in current studies. Medical research has confirmed that tissue regeneration and inflammation reduction occur after SVF therapy together with VEGF elevation and enhanced TGF-*β* production and decreased TNF-*α* and IL-1β pro-inflammatory markers. The intravenous SVF in degenerative joint disease is a study with increased VEGF levels after 2 weeks correlated with tissue vascularization (58). Angiogenic responses returned to normal levels following 8 weeks of observation. The results showed these responses to be brief but advantageous.

Efficacy evaluations are dependent on safety evaluations. Low adverse event rates, as reported in studies, are consistent. Most side effects from the procedure manifest as minor and temporary swelling or pain in the affected area before they disappear within several days. No systemic complications or immune reactions have been observed to date and SVF has been shown to be safe. Secondly, outcomes can be boosted by the synergistic effect of SVF in combination with PRP. Medical studies demonstrate that the combination of SVF and PRP leads to superior cartilage health outcomes and functional recovery in dogs as compared to either procedure used separately (83). They also found reduced from 67% pre-treatment to 26% at 6 months post-treatment NSAID use, but some cases saw a resurgence in NSAID use at 12 months (82). Therefore, the evaluation of SVF therapy for canine OA requires multiple assessment strategies between clinical observations of dogs and owner feedback together with biomechanical tests and imaging findings. The short-term benefits of the therapy are verified yet longer-term variations require additional research into optimal dosing protocols and multiple biomarkers to enhance treatment quality.

## 4 Comparison of SVF with other regenerative therapies

### 4.1 Comparison of SVF and adipose-derived stem cells

The regenerative properties of SVF and ADSCs have attracted attention in the therapeutic use in canine OA (60, 69). Both come from adipose tissue but are prepared differently, and possess different cellular compositions, functional characteristics, and clinical applications, all of which are important in this context in determining efficacy and feasibility.

#### 4.1.1 Composition and cellular heterogeneity

SVF is a heterogeneous cell mixture derived from adipose tissue via enzymatic di-gestion or mechanical disruption (65, 67, 88). It contains MSCs, endothelial cells and progenitors, pericytes, fibroblasts, and immune cells such as macrophages. The diversity allows SVF to have a multifaceted therapeutic effect, modulation of inflammation, angiogenesis, and tissue repair. However, ADSCs are isolated MSCs obtained through culture-expansion of cells extracted from SVF (89–91). However, these cells exist as a well-defined group with strong proliferative properties and also have the ability to convert into chondrocytes as well as osteoblasts and adipocytes. ADSCs separate from SVF through their lack of endothelial and immune factors while exclusively concentrating on stem cell treatment methods for regeneration.

#### 4.1.2 Preparation and practicality

Researchers process SVF from adipose tissue through the use of mechanical or enzymatic digestion methods with collagenase as the primary choice (67, 92). The process can be completed in a single day at the clinical site, making it a convenient “point-of-care” solution. It requires minimal equipment and no additional laboratory expansion. However, culturing and expanding MSCs from SVF in a process generally referred to as ADSC isolation requires sterile laboratory environments and several days to weeks. Although a more purified product with high cell yield is produced, they add time and cost as well as pose regulatory challenges to the process.

#### 4.1.3 Mechanisms of action

SVF contains a mixture of cells that exhibits multiple therapeutic mechanisms through immunological control alongside angiogenic effects and signaling between cells (63, 69). Macrophages and MSCs in SVF modulate pro-inflammatory cytokines like TNF-*α* and IL-1*β* while upregulating anti-inflammatory cytokine IL10. The function of tissue repair depends on the support provided by endothelial progenitor cells which enable vascular regeneration. The wide release of growth factors such as VEGF and TGF-β from SVF promotes cartilage repair as well as synovial tissue healing. However, ADSCs differentiate and exert a paracrine effect. The cells possess exclusive differentiation capabilities toward chondrocytes thus showing exceptional results in cartilage restoration. ADSCs also secrete bioactive molecules which are mainly focused on promoting cartilage matrix synthesis and reducing local inflammation.

#### 4.1.4 Efficacy in canine OA

The 2024 study simultaneously compares the efficacy of autologous SVF and allogeneic ADSC in the treatment of canine osteoarthritis (85). All dogs who received autologous SVF therapy showed instant improvements detected at Day 7 through reduced pain symptoms and gained range of motion along with the reduction of lameness scores (85). The treatment effects showed durability because subjects maintained or improved their benefits at the Day 30 follow-up evaluation. This suggests the use of SVF to support the healing of the joint in the future. SVF consists of regenerative cells in a rich variety and diverse contents including mesenchymal stem cells, endothelial progenitor cells, and immunomodulatory cells. This can preserve regenerative key factors in the fresh preparation of SVF and be superior to the activity of paracrine signaling and tissue repair. SVF treatment reveals effective results for all treated dogs especially when they have moderate to severe hip dysplasia.

However, the dogs that received allogeneic ADSC treatment experienced reduced clinical signs beginning on Day 7 as three out of five animals showed important reductions in pain symptoms and enhanced locomotor function (85). The therapeutic effects from the treatment kept progressing as four dogs demonstrated sustained improvement by Day 15 (85). The culture of ADSCs leads to increased potency and standardization for a homogeneous cell population with great differentiation and regenerative potential (60, 69, 89). Yet, the delay in preparation (culturing process) and absence of supporting stromal components may decrease its regenerative potential with respect to fresh SVF (60, 69, 85). At least one dog exhibited minimal improvement resulting from unrelated health problems and not the ADSCs themselves, a sign that ADSCs may work only if overall health is good and concurrent conditions also are good (85). The Day 30 results of ADSC treatment revealed similar regenerative therapy potential to SVF yet had slightly reduced consistency (85). When taken together, SVF has rapid and consistently good improvement with durable effects utilizing the advantages of fresh cells with diverse populations (60, 69, 85). Freshly isolated SVF demonstrates slightly superior effectiveness because it maintains its regenerative potential (60, 69, 85). Although ADSCs achieve steady regenerative advancement at a reduced speed than SVF, their standardized preparation methods show strong potential (60, 89–91).

#### 4.1.5 Practical considerations

The advantages of SVF treatment include its simple implementation process as well as its affordable price and broad delivery capabilities (69, 93, 94). It is processed and administered on the same day, which makes it very suitable for instant treatment. Requires less in terms of infrastructure than ADSCs. Targets multiple pathways due to cellular heterogeneity. The limitations of SVF are variability and short-term action. The inconsistent results arising from processing methods are due to the heterogeneous composition. Booster treatments may be required for effects to wane.

SVF’s minimal manipulation commonly avoids classification into stringent regulatory definitions, therefore being more clinically practicable (62, 95). ADSCs, due to their culture expansion, face more rigorous oversight as advanced therapy medicinal products (ATMPs) in many jurisdictions. Taken together, SVF and ADSCs bear complementary benefits for treating canine OA. The function and clinical potential of SVF treatment cover many disorders and simple application makes it easier to use in the clinic. ADSCs provide stronger and targeted regenerative benefits specific to their tissue of origin. A choice between the two depends primarily on the available resources, patient-specific needs, and clinical goals. A combination of therapies in many cases could optimize outcomes by affording the strengths of both therapies.

### 4.2 Comparison of SVF and exosomes

Therapies involving SVF and exosomes have potential as a regenerative treatment for canine OA (62, 96, 97). The two treatment modalities operate through separate biological paths that offer distinctive benefits and limitations in terms of mechanism structure and both financial considerations and practical usability and clinical effectiveness (94, 98, 99).

#### 4.2.1 Mechanistic and biological differences

SVF is a cellular approach. SVF is a heterogeneous mix of cells isolated from adipose tissue through enzymatic or mechanical methods (67, 95, 100). It contains MSCs, immune cells, endothelial cells and progenitors. MSCs perform two functions by differentiating into chondrocytes and by generating essential ECM elements needed for cartilage healing. They suppress pro-inflammatory cytokines such as IL-1*β* and TNF-*α* while promoting anti-inflammatory cytokines such as IL-10, and thereby they modulate the local inflammatory response. The process of angiogenesis gets enhanced through both endothelial cells and progenitors which establish new blood supply to injured tissues. Effects of SVF are exerting both direct cellular activity as well as secretion of bioactive molecules, such as growth factors (VEGF, TGF-β) (65, 100). The dual action repairs joints while simultaneously minimizing inflammation to boost joint movements. However, exosome therapy is a cell-free approach. Nano-sized extracellular vesicles secreted by MSCs and other cells are termed exosomes (101–106). Unlike SVF, the cell-free nature of exosome therapy depends on vesicular substances including microRNAs, proteins, and lipids that function to modify recipient cell activities. The major functions of exosomes include cartilage recovery together with immune regulation. Chondrogenic factors delivered by exosomes can promote the production of ECM (97, 102). The anti-inflammatory and anti-oxidative stress effects of specific microRNAs in exosomes contribute to OA suppression. Because exosomes do not need cellular engraftment, their activity is confined to paracrine effects. However, the process decreases both the probability of immune system rejection as well as tumor formation.

#### 4.2.2 Cost and feasibility

SVF is isolated at the point of care, using equipment available in many veterinary clinics (62, 95). The method begins with either enzymatic digestion or mechanical dissociation of cells that leads to subsequent centrifugation steps for cell concentration. The lower production costs make SVF a more attractive option for use in veterinary medicine on a much wider basis. The whole process can be done in a few hours so that same-day treatment is possible. However, the process of exosome isolation includes nurturing MSCs and collecting their released vesicles followed by product purification steps (101, 102). The procedure demands sophisticated laboratory equipment together with experienced personnel. However, the production and quality control are high in cost and therefore make exosome therapy less affordable, especially for smaller veterinary clinics. Unlike SVF which is composed differently based on isolation method and represents various products, exosome therapy is a reproducible and scalable product.

#### 4.2.3 Clinical outcomes

SVF medication results in considerable pain relief according to research which also leads to enhanced joint movement and fewer lameness incidents within short periods of time for dogs with osteoarthritis (56, 59, 62, 82, 95). SVF’s heterogeneity permits its use to improve OA’s inflammatory, vascular insufficiency and cartilage degradation aspects. The therapeutic effects of SVF usually sustain between 6 to 12 months before additional injections are needed for chronic disease patients. The length of treatment effect duration depends on both the seriousness of the disease and the procedures of intervention. The adverse side effects of SVF occur sparingly and are typically limited to mild swelling together with discomfort experienced where the injection occurs. The presence of live cells theoretically has a risk of uncontrolled differentiation or immune reactions, however, no severe cases have been reported. However, studies with living animals have shown that exosomes offer strong anti-inflammatory and healing powers (97, 102). The specific molecular signals they deliver effectively repair joint cartilage while also protecting it from more damage. Clinical data in veterinary applications are limited (107). A combination of factors makes exosome therapy that much more likely to experience longer-lasting benefits, and while they have very concentrated and potent molecular effects, we cannot say that with certainty until more longitudinal studies have been run and possible in clinical settings. Exosome therapy is cell free, and for that reason, it eliminates the possibility of tumor formation or immune rejection. As a result, it is a safer option, especially for systemic administration.

#### 4.2.4 Practical considerations

SVF consists of cellular activities with paracrine signaling as a regenerative mixture (62). It is simple, point-of-care produced can be used by most veterinary clinics, and is affordable (62, 95). SVF in all its forms is safe with only minimal adverse events and even rarer systemic risk (56, 62). Rapid onset of pain relief and improved mobility lasts 6–12 months, and such effects are evident clinically (56, 59). However, its efficacy has to be boosted over time by way of booster injections. In contrast, exosomes achieve therapeutic influence by using paracrine signaling exclusively (97, 102). These are complex to produce and need advanced facilities which makes the higher cost and limited adoption. Exosomes are extremely safe, with no cellular risks reported. The benefit of these types of therapy is that it holds promise for cartilage repair and anti-inflammatory effects, and the onset of those effects may not be as immediate as SVF (97, 102). Exosomes have the potential to yield longer-lasting effects, and research is ongoing to prove so and also to refine their use as a therapeutic tool.

Both SVF and exosomes may be exploited in combination: taking advantage of the immediate effects of applied SVF combined with the long-term molecular effects of exosomes (95, 97). The medical benefits from both therapies depend heavily on using standardized preparation protocols to allow complete reproducibility of treatment effects (62, 95). Reducing production costs for exosomes could expand their use in veterinary practice. SVF alongside exosomes functions as a different yet supportive method for treating canine osteoarthritis. The therapy using SVF demonstrates both affordability and flexibility as well as immediate accessibility. The targeted and permanent treatment quality of exosomes depends on standardized molecular actions. Future research and applications will benefit from combination strategies because these solutions provide a suitable option between the two treatments depending on clinical objectives, resource availability, and patient requirements (95, 97, 102).

### 4.3 Comparing SVF with other modalities

Canine OA has seen rising applications of regenerative treatments which now give veterinarians multiple management options for this disabling condition (62, 69, 95). In this way, SVF therapy is sometimes contrasted with other more modern regenerative processes including exosome, ADSCs, and PRP therapies (62, 69, 85, 94–96). The evaluation presents a detailed (Table 2) to provide practical guidance on mechanisms as well as cost-effectiveness and clinical outcomes and feasibility evaluation (95–97). SVF and exosomes may be used together in the future to utilize the immediate effects of SVF concomitant with the long-term effects of exosomes (95, 97, 102). PRP functions to increase the outcomes when used together with other methods (54, 82, 87). Consistency improvements along with enhanced clinical outcomes will result from developing one universal preparation method for SVF, exosomes along with ADSCs (67, 68, 95). The reduction of exosome and ADSC production costs will open new opportunities for modern therapeutic methods (95, 98, 99).

**Table 2:** Comparative analysis of regenerative therapies for canine osteoarthritis: mechanisms, cost, feasibility, efficacy, durability, and safety.

Criteria	SVF	ADSC	Exosome	PRP
Mechanisms	Combines cellular activity with paracrine signaling. It includes a mix of mesenchymal stromal cells, endothelial cells, and immune modulators, promoting inflammation reduction, angiogenesis, and cartilage repair.	A purified subset of MSCs isolated from SVF, ADSCs are known for their chondrogenic differentiation capabilities and potential to regenerate cartilage matrix.	Nano-sized vesicles delivering bioactive molecules such as microRNAs and proteins. These exert highly targeted effects on tissue repair and inflammation via paracrine pathways.	Derived from the patient’s own blood, PRP is enriched with growth factors that stimulate tissue repair and reduce inflammation. It is often used in combination with other therapies to enhance efficacy.
Cost	Moderate, as it can be processed on-site using enzymatic or mechanical methods.	Higher than SVF due to laboratory expansion and purification.	High, due to complex isolation and quality control processes.	Low to moderate, as blood is readily available and processing is straightforward.
Feasibility	Point-of-care preparation makes it widely accessible in veterinary clinics.	Needs specialized equipment and time for culture, which delays treatment.	Requires advanced laboratory facilities and expertise, limiting its widespread use.	Accessible and commonly used as an adjunct to other therapies.
Efficacy	Rapid improvements in joint mobility, pain relief, and lameness reduction. Its cellular heterogeneity addresses multiple OA pathologies.	Effective for cartilage repair due to their differentiation abilities but slower to act compared to SVF.	Promising preclinical results for cartilage regeneration and systemic inflammation control, but limited clinical data in veterinary applications.	Provides symptomatic relief and enhances the effects of therapies like SVF and ADSCs.
Durability	Benefits last 6–12 months, with booster injections needed for chronic cases.	Offers sustained cartilage regeneration but requires time to show measurable improvements.	Potentially longer-lasting due to concentrated bioactive molecules, though more studies are required.	Shorter duration of effect; often used as an adjunct for temporary relief.
Safety	Low risk of adverse effects; rare cases of mild swelling or transient discomfort.	Minimal side effects but require careful handling to prevent contamination or functional loss during expansion.	Very safe due to the absence of live cells, eliminating risks of immune rejection or uncontrolled differentiation.	Highly safe, as it uses the patient’s own blood. Rare instances of mild inflammation at the injection site.

SVF, Stromal Vascular Fraction; ADSC, Adipose-derived Stem Cells; MSCs, Mesenchymal Stem Cells; PRP, Platelet-rich Plasma; OA, Osteoarthritis.

## 5 Potential of SVF in combination with other treatment modalities

### 5.1 SVF and PRP combination therapy in canine osteoarthritis

The SVF, in combination with PRP, is now generating interest as a novel treatment for canine OA, as reported previously (61, 87, 108). The combined treatment capitalizes on PRP growth factors together with its anti-inflammatory elements as SVF enhances its natural restorative properties. This part provides an in-depth examination of how the therapy works with information about clinical applications and safety and efficacy results. Indeed, PRP is involved as a reservoir of growth factors and anti-inflammatory mediators in regenerative therapy. The regenerative functions of all of these growth factors delivered by PRP, such as VEGF, TGF-*β*, and platelet-derived growth factor (PDGF), are specific. Angiogenesis is stimulated and synovial fluid circulation is increased to enhance the overall joint microenvironment by VEGF. The activity of PDGF at the injury site draws both fibroblasts and MSCs to enhance tissue repair and TGF-β stimulates chondrocyte proliferation and matrix development for the purpose of cartilage regeneration. PRP is a substance derived from peripheral blood and is being investigated as a significant treatment option due to its pro-regenerative, as well as, anti-inflammatory effects, in that it reduces pro-inflammatory mediators, which slows cartilage degradation and can reduce joint pain. PRP when combined with SVF, leads to a very powerful effect since the two agents have complementary mechanisms. SVF cells strengthen the regenerative signals from PRP growth factors through an environment that helps cells survive and proliferate (87, 109). The united therapeutic power sets cartilage protection at a better level than individual treatments while promoting ECM reconstruction and recovering joint performance above separate treatment results. The combination approach creates a comprehensive therapeutic channel for joint pathology management that shows promise to develop better regenerative osteoarthritis treatments.

In 2024, a study of the long-term effects of a single intra-articular injection of autologous SVF and PRP combination in dogs diagnosed with elbow OA was evaluated (82). The implemented treatment yielded positive findings regarding clinical lameness scores while simultaneously reducing NSAID consumption until 6 months after administration. Some treated dogs also exhibited improvements in fore-hind limb symmetry which indicated enhanced gait motion patterns. Each dog showed different reactions to treatment and the treated group did not display uniform continuous benefits throughout follow-up. The treatment produced limited complications that included brief swelling and localized pain spread across only a few dogs but the study demanded bigger placebo-controlled tests. Such research would improve understanding of the efficacy and mechanisms of SVF-PRP therapy, and could, potentially, turn it into a standard therapy for OA in veterinary medicine. With this in mind, combined use of an SVF and a PRP may constitute a viable, minimally invasive alternative to current OA treatments to not only relieve symptoms in the affected dogs but also enhance their quality of life. According to the 2016 study, dogs who received both intra-articular and intravenous injections of autologous SVF and PRP simultaneously, reported a significantly decreased CBPI score (decrease in pain severity and decreased interference of pain to daily activities) at various intervals after treatment, and relatively better than single treatment cases (54). The treated dogs showed enhanced PVF outcomes especially when they had higher baseline functional deficits among the group serving as an indicator of better therapeutic results for severe cases of OA. The treatment maintained safety by showing only mild side effects including transitory leg discomfort or lameness that affected selected cases after injection. The data demonstrates that SVF along with PRP show potential as a minimal invasion therapeutic approach for OA management. The present animal investigative methods fail to accurately predict therapeutic success yet additional large-scale studies are needed to validate treatment results and reduce patient variance and refine therapy procedures across different levels of canine OA severity.

SVF and PRP application in regenerative therapy for OA has several limitations and challenges (109, 110). The major challenge regarding SVF and PRP treatment is standardization because scientists have not determined the best concentration ratios and diverse protocols lead to unpredictable therapeutic effects. Furthermore, PRP preparation is relatively inexpensive compared to SVF. In cases of such advanced OA with extensive cartilage damage, the SVF-PRP combination could perhaps be considered for adjuvant therapy to enhance the clinical benefit in situations where the SVF-PRP combination alone may not significantly reverse chronic degenerative changes. Although these challenges remain, there are many promising avenues to look ahead at in order to improve the efficacy of SVF-PRP therapy. The therapeutic strategy receives increased effectiveness when combined with adjunctive treatments such as hyaluronic acid injection or anti-inflammatory medications (70, 86). Standardization and automation of the SVF and PRP preparation systems may lead to a reduction of the cost of such therapies and improve consistency, increasing availability. Moreover, it is necessary to extend research with further longitudinal studies to confirm the durability of results and to fine-tune protocols of given canine populations and OA stages. Future development is essential to achieve precise clinical implementation of SVF-PRP therapy in veterinary regenerative healthcare.

### 5.2 Potential of SVF with NSAID therapy in canine osteoarthritis

Combine SVF with NSAIDs and this approach shows promise for treating canine OA (3, 42, 111). The benefit of this integration is that it takes care of both symptomatic relief management and underlying joint degeneration, thereby providing a dual benefit to the overall treatment outcome. Combining SVF therapy with NSAID therapy will represent a synergistic approach that could be used for treating canine OA. Immediate pain relief produced by NSAIDs occurs by inhibiting cyclooxygenase (COX) enzymes and the inevitable elimination of inflammation (44, 112). However, there are multiple NSAIDs including carprofen along with meloxicam and robenacoxib that are effective pain medications yet they result in long-term side effects that affect the gastrointestinal system and kidneys (113–115). By contrast, SVF’s tissue restorative qualities derive from MSCs together with additional factors that control immune system functions and trigger tissue repair mechanisms. The MSCs contained in SVF produce anti-inflammatory cytokines such as IL-10 that block pro-inflammatory mediators such as TNF-*α* and IL-1β, from OA genesis. Combining NSAIDs and SVF provides the clinical benefit of better symptom management, less long-term NSAID dependency, and better overall joint health. NSAIDs provide rapid pain relief, while SVF decreases synovial fluid inflammatory biomarkers due to their anti-inflammatory effect and nutritional, trophic factors, and growth factors, which regenerate cartilage. This dual approach has been demonstrated in studies to show better mobility, less numbing of pain scores, and improved cartilage integrity as opposed to NSAID monotherapy (82). In addition, the requirement for long-term NSAID use can be reduced, thereby avoiding related risks. By way of example, the protocol may include NSAIDs as always around as fast as needed be pain relieving and then intra-articular injections with SVF, which might average 2–10 million viable cells per joint. The booster doses may also be given at 6–12 month intervals as required. Safety profiles are favorable for both SVF and reduced adverse effects from NSAIDs secondary to lower dosages. SVF therapy shows potential as an extended OA treatment despite its cost considerations because it offers healing capabilities and integrates well with NSAIDs therapy.

### 5.3 Potential of SVF with nutraceuticals in canine osteoarthritis

The combination use of SVF and nutraceuticals provides a total approach to treating canine OA (40, 48). SVF’s native healing potential is enhanced through joint forces with the biochemical nutraceuticals like glucosamine and chondroitin sulfate alongside omega-3 fatty acids and green lipped mussel extract. This evaluation explores the supportive benefits between the treatment approaches alongside their observed clinical results along with identified concerns. Glucosamine and chondroitin sulfate are building blocks for cartilage ECM synthesis and inhibit cartilage degradation by preventing MMPs (46, 116–120). The inflammatory pathways maintain lower activity levels when patients consume EPA and DHA omega-3 fatty acids because these acids decrease PGE2 and leukotriene levels while changing synovial fluid composition. The combination of anti-inflammatory and antioxidant effects of Green-lipped mussel extract reduces oxidative stress on cartilage (121, 122). SVF acts as a cellular regenerator while nutraceuticals supply the biochemical elements needed for cartilage healing in a combined manner. The anti-inflammatory actions of SVF receive additional support from omega-3 fatty acids combined with green-lipped mussel extract to enhance the regulation of inflammatory mediators present in the joint.

Glucosamine and chondroitin sulfate are among the nutraceuticals that promote SVF stimulation of chondrocyte activity and ECM synthesis (114). SVF and nutraceuticals merge their approaches by providing structural and biochemical solutions to cartilage healing problems (114, 117). SVF reduces joint inflammation together with omega-3 fatty acids which results in effective pain relief (115). The dual mechanism of therapy works well for chronic OA patients experiencing long-term inflammation (115, 117). SVF’s regenerative effects are complemented by green-lipped mussel for its ability to lubricate synovial fluid. Reduction of joint friction combined with enhanced mobility results from the use of this synergy therapy. The combination of SVF with nutraceuticals may slow the progression of OA, by reducing cartilage degradation markers and oxidative stress, thus preserving joint health over the long term (117). Therefore, glucosamine and chondroitin sulfate are typical oral dosage such as 15–30 mg/kg/day for dogs, divided into two doses (118). The omega-3 fatty acids are given within the dosage of 50–75 mg of EPA/DHA per kg of body weight daily (118). The dosage of green-lipped mussel extract is approximately 20–50 mg/kg/day depending on the formulation (118). The periodic treatment from SVF functions for cellular regeneration but patients need daily nutraceuticals for biochemical maintenance (114, 117). Clinical signs and joint biomarkers are regularly monitored to ensure the need for adjustment in dosage or timing (117, 119). Most nutraceuticals show good tolerance along with few associated adverse effects (117). Glucosamine and omega-3 high doses can result in light stomach discomfort (118). The combination of SVF and nutraceuticals minimizes the pharmaceutical drug use of NSAIDs to reduce possible long-term negative side effects (123). Long-term administration of nutraceuticals, however, requires owner compliance to continue to provide benefits. When combined, SVF is also utilized with nutraceuticals supporting both cellular regeneration and biochemical support in the management of canine OA. This combination improves cartilage repair, decreases inflammation slows disease progression, and is a great option for long-term joint health.

### 5.4 Potential of SVF with physical therapy in canine osteoarthritis

The joint reconstruction potential of SVF cells when coupled with available physical therapy makes them a robust multiple-healing solution for canine OA. The system integrates SVF cells’ regenerative capabilities with physical therapy benefits through therapeutic ultrasound and ESWT treatments as well as exercise therapy (47, 50, 124). Combining this together produces synergistic effects which overall help joint health, help dogs move better, and take that pain away. Therapeutic ultrasound (TU) enhances tissue healing by boosting blood flow along with tissue metabolism rates and accelerates chondrocyte cell healing and soft tissue repairs through mechanical vibrations and heat applications (125). Experimentally activated shock waves used in ESWT produce positive effects on both nerve perception and inflammatory response while also promoting tissue repair and neovascularization through microtrauma generation (126). Exercise-based rehabilitation improves joint stability and strength by enhancing muscle support around affected joints and reduces stiffness and maintains range of motion through controlled movement (127, 128).

The combination of SVF with TU and ESWT creates an accelerated process through which SVF cells integrate effectively thus leading to better joint repair. ESWT and TU achieve pain reduction through control of nerve signals alongside inflammatory mediators. Thanks to their combined anti-inflammatory properties and pain relief mechanisms SVF reduced the need for drug dosages while shortening the time until pain relief while extending treatment duration thus improving dog patient comfort. Good physical therapy will restore muscle strength, flexibility and complement the structural repair initiated by SVF, and improve mobility. The enhanced circulation through TU improves synovial fluid health by decreasing joint friction thus helping SVF perform additional regeneration to improve joint health. In addition, ESWT also stimulates vascularization creating additional benefits for synovial joint health which extends to long-term joint well-being. Therefore, therapeutic ultrasound uses frequency around 1–3 MHz, applied for 5–10 min per session and schedule around 2–3 sessions per week for 4–6 weeks (51). Extracorporeal shockwave therapy uses frequency around 1–3 treatments at 2–3 week intervals and intensity of energy levels between 0.03–0.25 mJ/mm^2^ to avoid excessive discomfort (50). Exercise-based rehabilitation consists of regular activities which include movements to increase range of motion and underwater treadmill sessions in combination with low-impact strength exercises. The rehabilitation programs for high-severity diseases use custom-made approaches. Exercise effectively prevented the development of joint instability together with additional cartilage damage by sustaining joint stability and preserving muscle mass (129, 130). Additionally, TU and ESWT are generally well-tolerated. ESWT may cause mild discomfort during application but subsides quickly. Herein considered together, the addition of SVF to physical therapy creates a synergistic framework for treating canine OA. SVF completes the biomechanical effects achieved by TU and ESWT alongside exercise-based rehabilitation programs. Joint function enhancement occurs when medical professionals combine these treatment methods while improving both manual strength and comfort of movement.

## 6 Challenges in the clinical application of SVF for canine osteoarthritis

### 6.1 Standardization and quality control

Therapeutic use of canine SVF in OA requires the process of preparation to be rigorously standardized to ensure consistent results, reduce variability, and maintain safety standards. Standardization and quality control contain several essential components such as fat site selection, donor variability and processing techniques, cell quality evaluation and storage and dosing consistency, and regulatory requirements and compliance in addition to their respective difficulties and limitations. (1) The acquisition of fat tissue must first consider two factors, such as site selection and donor variability. It is well recognized that the yield and quality of SVF cells are very much dependent on the adipose tissue collection site. Subcutaneous abdominal fat, falciform ligament fat, and peri ovaries fat display different forms of impact on the quality and quantity of the SVF cells (131). Subcutaneous abdominal fat yields the lowest amount of SVF cells (4.18 ± 8.25 × 10^6^ cells/g) and requires extensive dissection, with moderate cell viability (94.94% ± 2.9%) and surface marker expression (60). Falciform ligament fat offers a higher adipose tissue volume but lower SVF purity, as evidenced by lower CD90^+^ expression (17.65 ± 5.52%) and reduced CD45-hematopoietic stem cell exclusion (70.35 ± 6.33%). In contrast, peri-ovarian fat stands out as the premier source because it yields the maximum SVF quantity (36.87 ± 19.6 × 10^6^ cells/g) coupled with outstanding cell viability (99.63% ± 0.2%) and high expression of mesenchymal stem cell indicators CD90, CD44, and CD29 for regenerative treatments (60). (2) Variation in donors poses a direct effect on the quality of the harvested cells to be used for treatment. Some parameters such as age are critical since the cells isolated from young animals are more efficient, producing a larger number of viable cells with higher differentiation potential than their elderly counterparts (60, 132). Likewise, cell viability is affected by spay/neuter status. Non-spayed dogs provide more viable cells, presumably due to hormonal effects on adipose tissue. Also, factors including obesity and metabolic syndromes affect the quality of cells by producing pro-inflammatory conditions that lower the effectiveness. To overcome these problems, the following recommendations should be considered: to set the criteria for the selection of the donors according to their age, health status, and fat deposit location, as well as to design special protocols for different types of tissues in order to achieve the highest effectiveness of cell isolation and viability.

(3) Processing methods for isolating SVF involve two main approaches: These are chemical isolation with enzymatic isolation, and mechanical isolation all with pros and cons. Although higher numbers of viable cells are obtained through enzymatic isolation, using collagenase as the gold standard, this method has limitations associated with the use of enzymes, which would require regulatory approval and may lead to residue in the final preparations (55, 67). Risks associated with enzymatic activities during stem cell isolation are minimized with mechanical isolation approaches such as filtration and centrifugation although these methods produce reduced cell yields and viability statistics (55, 67). Mechanical cell separation methods enable easy operation and provide mobile healthcare solutions and match regulatory criteria for minimally altered medical procedures. Combinations of enzymatic and mechanical methods for cell processing are suggested to optimize cell yield and increase compliance to standardize processing equipment such as centrifuges speeds and filtration pore size for reproducibility and efficiency. (4) Establishing quality parameters of cells is critical to the therapeutic efficacy of SVF. Thus viability testing is a crucial step and cells with a viability over 80% prior to administration are needed (54). Basic techniques including Trypan blue exclusion or more advanced techniques such as flow cytometry in order to analyze particular populations of cells can be used to assess this. The therapeutic characteristics of SVF can be evaluated through marker expression tests that focus on MSC markers CD90 together with CD73 alongside assays for quantifying endothelial and immune cells (80). Every clinic should use standardized viability and marker expression testing methods uniformly to predict therapeutic outcomes better before therapy administration. (5) The shelf-life extension technique of SVF depends on cryopreservation since this process enables both single-use and multiple administration of products at room temperature. Freezing and thawing procedures face difficulties in cell preservation and ice crystal prevention so the standard solution includes applying DMSO as a cryoprotectant. The therapeutic quality of frozen cells requires proper evaluation of cooling speed alongside accurate thaw temperature alongside post-thaw cell viability and functionality testing before clinical implementation (60). (6) One key challenge in SVF therapy is to do a dosing consistency due to variability in cell concentration and composition (59). The therapeutic cell dose remains undecided so healthcare providers administer between 2 and 10 million cells into each joint while increased cell concentrations tend to provide better results. Cell composition variability in SVF creates additional complexity for standardization procedures. A study needs to establish minimal effective and optimal dosage levels through clinical trials while devices need to allow automated cell counting and concentration adjustment for consistent therapeutic results. (7) Safety and compliance are important in SVF therapy, as there needs to be stringent measures to ensure sterility and regulatory standards. Closed system processing devices decrease exposure risks, and sterility must be ensured throughout all stages of SVF preparation to prevent microbial contamination and endotoxin presence. Various regulatory institutions define SVF as a minimally processed substance and enforce processing limitations while establishing precise documentation and method requirements (55). To overcome this, it is suggested to include sterility testing before administration, and the protocol is harmonized with the local regulatory framework to abide by compliance and patient safety. (8) Challenges to SVF therapy include but are not limited to, lack of standardization, therapeutic consistency, and cost. Therefore, the conflicting isolation methods together with equipment and reagents cause SVF preparations to become different from one another which reduces the reliability and clinical effectiveness. The therapeutic results of SVF are influenced by inconsistent SVF composition which includes the MSCs to non-MSCs percentage and immune cells’ number (60). Standardized processing equipment together with necessary consumables proves too expensive for veterinary practices to afford especially those with limited size. The implementation of SVF therapy requires a solution to existing challenges so that it becomes more widely usable. Standardization of SVF preparation and the development of a robust, quality control system is important to guarantee consistent clinical outcomes in the use of canine OA therapy. Addressing variability in tissue harvesting, isolation methods, and cell assessment will enable researchers and clinicians to use the most effective SVF therapies safely, with the best chance of efficacy and reproducibility.

### 6.2 Long-term effects and safety considerations

The treatment shows positive outcomes for repairing degenerative joint conditions in dogs with OA. Nevertheless, the long-term effects and safe use of this treatment option need to be addressed to improve clinical outcomes on a more permanent basis. SVF therapy is promising both in long-term efficacy and poses challenges. Studies show that one dose of SVF injections produces notable pain relief together with functional benefits between 3 to 6 months yet these effects mostly fade by 12 months particularly when dealing with advanced cases of OA in which long-term cartilage regeneration proves elusive (59, 82). Dog elbow joint treatment with SVF injections gradually improved range of motion abilities and weight-bearing capacities while multiple dogs needed second treatments because symptoms returned within a one-year period (59, 82). The administration of greater than 10 million viable cells per injection appears to show better outcomes for patients with moderate-stage OA but advanced cases demonstrate limited sustained results and fast progression that supports the identification and treatment of patients based on disease severity (54). MRI scans alongside other assessments reveal that cartilage tissue generates in select cases yet the mechanical characteristics of new tissue remain inferior to pristine cartilage with recurring symptoms (133, 134). Long-term evaluations need advanced imaging or arthroscopy tests in order to monitor tissue quality while attempting to enhance therapeutic durability.

Several critical aspects are involved with safety considerations in SVF therapy. Immunogenicity and immune reactions are minimized by the immune-modulating properties of SVF, though allogeneic SVF may have a possibility to trigger antibody formation, with repeated injections heightening the risk of immune responses, necessitating careful patient monitoring. Strict controls must be established due to the potential tumorous properties of SVF and MSC cells despite their concern because persistent joint inflammation could transform into malignancy (60). The outcome is patient-specific and is also dependent on the age, weight, and metabolic health of the patient with obese dogs with metabolic syndrome or older dogs with reduced MSC regenerative capacity showing less good outcomes. The rate of infection together with anesthesia-related issues decreases when using minimally invasive approaches although this results in lower cell production levels. Standardized protocols for harvesting and processing would improve both safety and consistency. The current research period is insufficient because most trials operate for 6–12 months which prevents the detection of delayed adverse effects and reduced therapeutic outcomes. Validated scoring systems and advanced imaging techniques such as MRI must be used for long-term follow-up to fully assess the effects of therapy and to protect the patient.

Both Major and minor complications are potential risks in SVF therapy. Local swelling together with joint effusion along with short-term pain exist as manageable minor issues although major systemic responses and infections remain very rare among both canine and human trials (54). Manufacturing repeated for all the SVF therapies is still in-consistent and the quality control between these clinics is relatively varied (60). Safety concerns can be mitigated through adherence to good manufacturing practices (GMP) and the requirement of strict quality assurance programs. In addition, safety evaluation becomes more complex due to combined therapy protocols that integrate SVF cells with other biological treatments such as PRP to stimulate cartilage reconstruction, demonstrating the requirement for standardization of protocols. In the future, placebo-controlled randomized trials over long periods are needed to demonstrate that SVF is safe and effective in the long term (82). However, minimally invasive approaches for procuring cells along with optimized processing techniques will achieve better outcomes while reducing safety risks. Novel add-on therapies which include anti-inflammatory drugs and advanced imaging platforms could establish new means to enhance treatment reliability and security (103). The adoption of SVF therapy as a standardized and safe treatment for canine osteoarthritis remains possible when challenges are met which will yield long-term benefits yet minimal risks.

## 7 Future perspectives of SVF in canine osteoarthritis treatment

### 7.1 Future research directions

Despite great promise, more exploration must occur to refine the therapeutic application of SVF to canine OA to ensure both long-term and maximal efficacy and address the hurdles associated with its use. The therapeutic dosages of SVF therapy require optimization since reported cell counts in injections range from 2 to 10 million cells yet the link between cell concentration and treatment effectiveness remains unclear (56). To address this challenge, dose–response studies need to be undertaken to discover the minimum effective dose with appropriate cost efficiency. Furthermore, patient-specific factors, such as age, body weight, and disease severity can be also utilized to tailor the dosing strategies. In addition, the dosing strategies for SVF implementation can be adapted by using individual patient factors like disease severity alongside age and body weight. An exploration of alternative administration routes for SVF therapy should enhance its utility and efficacy (54). Systematic delivery, as in intravenous delivery, may benefit dogs with multi-joint osteoarthritis, because they can achieve anti-inflammatory effects at remote sites, while most intra-articular injections are the most commonly used approach. Future studies should evaluate and compare the effectiveness of intra-articular, intravenous, and combined administrative procedures through controlled clinical tests. Furthermore, innovative means of delivery, like magnetic guidance or receptor-based targeting, could be used to improve homing of SVF cells to target tissues for maximum therapeutic benefits. This is essential to overcome current limitations for enhancing long–term monitoring and efficacy in SVF therapy (59). However, studies have short follow-up periods (6–12 months) and subjective assessments of treatment effects, leading to problems with evaluating the durability of therapeutic effects and introducing bias. Advanced imaging modalities such as MRI and ultrasound elastography will objectify monitoring of cartilage regeneration and of the joint condition, and biomarkers of inflammation and tissue damage, as well as collagen turnover markers, will inform on biological response. Outcomes assessment across multiple years together with the persistence of therapeutic benefits and booster treatment requirements will need longitudinal research studies for complete evaluation.

The combination strategies involving SVF show great promise to produce enhanced treatment results for OA individuals. The application of SVF together with PRP demonstrates the potential to achieve increased anti-inflammatory and regenerative benefits whereas additional medicines such as hyaluronic acid or disease-modifying osteoarthritis drugs may support better OA treatment (82). Future medical research needs to determine how different combined treatments should be sequenced and dosed as well as evaluate their economic value and practicality for veterinary medicine. Standardizing SVF therapy remains essential because it will help reduce treatment variations which result in inconsistent medical results. As SVF consists of a heterogeneous mixture of MSCs, endothelial cells, immune cells, and growth factors, SVF processing can be variable between patients as there are large differences in the preparation methods used. Developed standard protocols must optimize cell quantity together with cell survival rates and research consistency in order to reduce this problem. Consistent product quality in all clinics depends on strict quality control protocols which include cell viability assessments and phenotypic cell evaluations. Furthermore, the influence of donor factors such as age and body condition score on the therapeutic quality of SVF should be investigated to aid with efforts to standardize therapies. A precision medicine approach to SVF therapy will improve efficacy since it matches treatments specifically for each dog with OA. Dogs presented with different degrees of the progression of disease, comorbidities, and responses to treatment, therefore, personalized strategies are needed. This proposal shows how biomarkers can help classify patients based on their inflammatory status as well as cartilage damage levels and tissue healing potential to forecast treatment responses. Furthermore, the integration of machine learning models for the analysis of patient-specific data can predict the best treatment regimen for a specific patient thus promoting personalized veterinary care. Although SVF therapy is mainly investigated for the treatment of OA, its utilization has been extended to other orthopedic and systemic diseases (54, 58, 85). Research on SVF therapy should focus on three main areas: tissue regeneration after ligament tears, the treatment of intervertebral disc disease and systemic inflammatory diseases and avoidance of fibrosis during surgical recovery. Adopting SVF therapy at its full potential requires scientists to study methods for delivering proper doses together with sustained monitoring of medical outcomes. The veterinary management of OA can undergo a transformation through the development of advanced diagnostic methods and customized treatment plans and additional therapeutic methods. The future demands researchers should work together with bioengineers and veterinarians to advance current SVF limitations so the therapy can achieve its maximum potential.

### 7.2 Integration of advanced technologies

The great potential of innovative technologies to improve SVF therapy, and thus, serve as the future therapy for canine OA exists. The promising characteristics of SVF therapy are enhanced through new advanced techniques for safety and scalability and cell engineering and gene editing and advanced bioprocessing technologies. (1) The gene editing approach using CRISPR-Cas9 changes the way MSCs function inside the SVF by making precise site-specific genetic changes (79, 110). Genetically engineered enhancements that stimulate TGF-*β* and BMP-7 expression lead to better cartilage healing while minimizing the production of pro-inflammatory cytokines IL-1β and TNF-*α* and total body inflammation. The activation of telomerase extends MSC life span and regenerative properties so they can overcome physiological deterioration that occurs with age. The altered expression of immune-related genes such as HLA through gene editing allows therapists to decrease tissue rejection in allogeneic applications and provides patients with anti-inflammatory mediators like IL-10 to enhance their joint environments. Moreover, disease-specific customization can enhance treatment for breeds that are prone to osteoarthritis including the Labrador Retriever dog breed or the German Shepherd dog breed, adjusting therapies for genetically predisposed susceptibilities in order to obtain desirable outcomes. (2) Cell engineering intends to optimize the cell function of SVF-derived cells for improved adaptation to osteoarthritic microenvironment. Hypoxic preconditioning reproduces joint hypoxic conditions for cells and strengthens their survival potential along with their regenerative capacity. Mechanical preconditioning exposes cells to simulated joint stresses to prepare them for the mechanical demands of the environment (61, 79). The combination of SVF cells with bioengineered scaffolds delivers two functions: optimal cell spread and bioactivity provided by embedded growth factors and anti-inflammatory agents (135). The combination of paracrine enhancement technology enhances regenerative healing through exosome and extracellular vesicle secretion containing cartilage-regenerating microRNA such as miR-140 as well as targeted factor production such as VEGF and IGF-1 to improve both angiogenesis and cartilage regeneration. Research into standard procedures for SVF therapy has expanded its benefit potential for osteoarthritis patients. (3) There exists a fundamental change in SVF preparation and application resulting from novel bioprocessing technologies that provide standardized and efficient scalable processes. Standardized isolation and concentration of SVF are possible in automated processing platforms, including point-of-care devices capable of automated cell counting and viability assessment improving quality control (60). Expanding the SVF-derived cells with bioreactor technologies under controlled conditions, with dynamic cultures simulating the joint environment to precondition the cells for better therapeutic outcomes is proposed. Furthermore, delivery advances including nanoencapsulation help protect SVF cells for targeted delivery and slow release within the joint, as well as application of an injectable hydrogel to maintain cell retention and activity at injury sites raise the effectiveness of SVF therapy. The findings generated from this research improve bioprocessing methods to enable better treatment delivery of consistent and effectual therapies. (4) Computational tools aiming at SVF therapy can be greatly strengthened with artificial intelligence. Using predictive machine learning models patient-specific information about age, breed, weight and OA severity helps analyze therapeutic outcomes and determine appropriate dosage levels and administration methods among systemic treatments (136). Wearable devices with AI-powered monitoring functions enable real-time tracking of patient recovery movements as well as pain metrics through feedback to assess treatment effectiveness on the spot. Such extended monitoring allows clinicians to reevaluate therapy protocols as needed for maintaining stable and unique treatment responses in real-time. SVF shows promise as a breakthrough therapeutic for regenerative medicine applications when utilized with advanced tissue engineering and genetic therapies to treat OA. SVF can function as a vector to deliver therapeutic genes for gene therapy, allowing modification of the joint environment in order to promote sustained cartilage regeneration. The medical procedures for joint resurfacing benefit from engineered tissue constructs including bioengineered cartilage grafts which use SVF cells as a source for innovative treatment of complex conditions. Pre-engineered SVF cells stored in allogeneic cell banks present potential as off-the-shelf standard therapeutic cells to meet current access and operational requirements. The introduction of gene editing and cell engineering together with bioprocessing integration to SVF therapy methods will generate a new standard for effective and safer canine OA management by enhancing both product effectiveness and operational scalability.

### 7.3 Expanded clinical prospects

SVF shows massive importance for both veterinary medicine and human regenerative medicine beyond orthopedic applications such as canine. Additional insights from canine OA SVF applications can be used to explore new classes of therapeutics and approaches for human OA since canine and human OA share substantial pathophysiology and therapeutic responses. In addition to bone injuries, SVF also shows promise for the treatment of other veterinary conditions including injuries to soft tissues, intervertebral disc disease and ligament damage, all of which increase the role of SVF in overall veterinary care. SVF is a bridge between veterinary and human regenerative medicine and produces progression in both fields primarily by soaking up the achievements of the other. SVF demonstrates promise in veterinary orthopedics which presents an opportunity to create broad-ranging effects through the future thus shifting OA and other OA-related conditions from symptom management to tissue renovation. New processing technologies are promising lower-cost, standard full SVF treatments that could be globally available which is good news for canine patients everywhere (60).

## 8 Conclusion

SVF therapy has become an important breakthrough in canine OA due to the ability to improve and counter the complex pathophysiology of this debilitating disease on several fronts (54, 56, 59, 62). The presence of MSCs together with endothelial progenitor cells along with immune-modulating cells in SVF enables cartilage regeneration while supporting blood vessel health and showing anti-inflammatory properties for the treatment and healing of OA. Clinical tests demonstrate that SVF treatments through the combination of PRP and hyaluronic acid provide swift medical benefits including improved joint function with reduced pain together with enhanced patient well-being. The fact that it is practical, able to be processed on the day, at the point of care, makes it attractive to the veterinary practitioner. However, the full therapeutic potential of SVF needs clinical practice implementation through supportive methods. The effectiveness of SVF depends on patient selection criteria because it works best in early to moderate OA cases yet additional treatments show better results with advanced stages while most successful results arise from early intervention. Intra-articular injections and the determination of a specific dosage are administered following standardized administration protocols to guarantee reliable results. SVF is combined with physical rehabilitation to accelerate recovery and promote long-term joint function as we provide longitudinal monitoring to allow follow-ups and booster treatments when needed.

Although current applications illustrate the potential of SVF it will not soon reach its role in veterinary medicine without additional research. The field needs consistent practices for making SVF and delivering it to patients while long-term tests confirm safety and effectiveness plus researchers may use new technology like genetic modification tools. Moreover, other conditions, including intervertebral disc disease and ligament injuries, will diversify the clinical applications of SVF. Economic accessibility remains vital for development since growth of utilization in underprivileged locations depends on it (95, 98). SVF therapy brings modern innovations that combine with the current operational functionality to join contemporary technologies with upcoming developments. This capacity to regenerate damaged tissue, modify inflammation, and also to improve quality of life qualifies it as a central pillar for regenerative veterinary care. Embracing SVF, veterinary clinicians can increase the outcomes for canine patients, and participate in the creation of the future of regenerative medicine. The ongoing commitment to research and clinical application alongside collaboration will enable SVF to achieve a breakthrough in OA treatment as it transforms pet and human lives.

## Glossary

OA: Osteoarthritis
SVF: Stromal vascular fraction
ECM: Extracellular matrix
MMPs: Matrix metalloproteinases
IL-1*β*: Interleukin-1 beta
TNF-*α*: Tumor necrosis factor-alpha
CBPI: Canine brief pain inventory
MRI: Magnetic resonance imaging
CT: Computed Tomography
COMP: Cartilage oligomeric matrix protein
CTX-II: C-terminal telopeptide of type II collagen
NSAIDs: Non-steroidal anti-inflammatory drugs
ESWT: Extracorporeal shockwave therapy
MSCs: Mesenchymal stem cells
ADSCs: Adipose-derived stem cells
VEGF: Vascular endothelial growth factor
TGF-β: Transforming growth factor-beta
DMSO: Dimethyl sulfoxide
EPCs: Endothelial progenitor cells
Treg: Regulatory T cells
HSC: Hematopoietic stem cells
IA: Intra-articular injection
IV: Intravenous
IL-10: Interleukin-10
PGE2: Prostaglandin E2
NF-κB: Nuclear factor kappa B
IGF-1: Insulin-like growth factor-1
FGF: Fibroblast growth factor
PRP: Platelet-rich plasma
PLGA: poly(L-lactide-co-glycolide)
VI: Vertical Impulse
HVAS: Hudson Visual Analogue Scale
ATMPs: Advanced therapy medicinal products
PDGF: Platelet-derived growth factor
COX: Cyclooxygenase
TU: Therapeutic ultrasound
GMP: Manufacturing practices
PVF: Peak Vertical Force

## References

[1] Brondeel C Pauwelyn G de Bakker E Saunders J Samoy Y Spaas JH. Review: mesenchymal stem cell therapy in canine osteoarthritis research: "Experientia Docet" (experience will teach us). Front Vet Sci. (2021) 8:668881. doi: 10.3389/fvets.2021.668881, PMID: 34095280 PMC8169969

[2] van den Berg WB. Lessons from animal models of osteoarthritis. Curr Opin Rheumatol. (2001) 13:452–6. doi: 10.1097/00002281-200109000-00019, PMID: 11604604

[3] Pye C Bruniges N Peffers M Comerford E. Advances in the pharmaceutical treatment options for canine osteoarthritis. J Small Anim Pract. (2022) 63:721–38. doi: 10.1111/jsap.13495, PMID: 35285032 PMC9790257

[4] Michelsen J. Canine elbow dysplasia: aetiopathogenesis and current treatment recommendations. Vet J. (2013) 196:12–9. doi: 10.1016/j.tvjl.2012.11.009, PMID: 23266351

[5] Wang Y Chen Y Wei Y. Osteoarthritis animal models for biomaterial-assisted osteochondral regeneration. Biomater Transl. (2022) 3:264–79. doi: 10.12336/biomatertransl.2022.04.006, PMID: 36846505 PMC9947734

[6] Xia B di Chen Zhang J Hu S Jin H Tong P. Osteoarthritis pathogenesis: a review of molecular mechanisms. Calcif Tissue Int. (2014) 95:495–505. doi: 10.1007/s00223-014-9917-9, PMID: 25311420 PMC4747051

[7] Ratneswaran A Rockel JS Kapoor M. Understanding osteoarthritis pathogenesis: a multiomics system-based approach. Curr Opin Rheumatol. (2020) 32:80–91. doi: 10.1097/BOR.0000000000000680, PMID: 31724972

[8] Jimenez G. Osteoarthritis: trauma vs disease. Adv Exp Med Biol. (2018) 1059:63–83. doi: 10.1007/978-3-319-76735-2_3, PMID: 29736569

[9] Wei Y Bai L. Recent advances in the understanding of molecular mechanisms of cartilage degeneration, synovitis and subchondral bone changes in osteoarthritis. Connect Tissue Res. (2016) 57:245–61. doi: 10.1080/03008207.2016.1177036, PMID: 27285430

[10] Scanzello CR Goldring SR. The role of synovitis in osteoarthritis pathogenesis. Bone. (2012) 51:249–57. doi: 10.1016/j.bone.2012.02.012, PMID: 22387238 PMC3372675

[11] Molnar V Matišić V Kodvanj I Bjelica R Jeleč Ž Hudetz D. Cytokines and chemokines involved in osteoarthritis pathogenesis. Int J Mol Sci. (2021) 22:9208. doi: 10.3390/ijms22179208, PMID: 34502117 PMC8431625

[12] Wang L Xu H Li X Chen H Zhang H Zhu X. Cucurbitacin E reduces IL-1beta-induced inflammation and cartilage degeneration by inhibiting the PI3K/Akt pathway in osteoarthritic chondrocytes. J Transl Med. (2023) 21:880. doi: 10.1186/s12967-023-04771-7, PMID: 38049841 PMC10696753

[13] Vincent TL. Mechanoflammation in osteoarthritis pathogenesis. Semin Arthritis Rheum. (2019) 49:S36–8. doi: 10.1016/j.semarthrit.2019.09.018, PMID: 31779850

[14] Zeng CY Zhang ZR Tang ZM Hua FZ. Benefits and mechanisms of exercise training for knee osteoarthritis. Front Physiol. (2021) 12:794062. doi: 10.3389/fphys.2021.794062, PMID: 34975542 PMC8716769

[15] Yu G Segel I Zhang Z Hogan QH Pan B. Dorsal root ganglion stimulation alleviates pain-related behaviors in rats with nerve injury and osteoarthritis. Anesthesiology. (2020) 133:408–25. doi: 10.1097/ALN.0000000000003348, PMID: 32433276 PMC8195267

[16] Thoene M Bejer-Olenska E Wojtkiewicz J. The current state of osteoarthritis treatment options using stem cells for regenerative therapy: a review. Int J Mol Sci. (2023) 24:8925. doi: 10.3390/ijms24108925, PMID: 37240271 PMC10219560

[17] Rychel JK. Diagnosis and treatment of osteoarthritis. Top Companion Anim Med. (2010) 25:20–5. doi: 10.1053/j.tcam.2009.10.005, PMID: 20188335

[18] Oehme S Milinkovic DD Paolucci A Krafzick S Fahy S Damm P. Autologous bone grafting combined with spheroid-based matrix-induced autologous chondrocyte implantation for osteochondral defects of the knee: good clinical outcomes alongside abnormal postoperative gait patterns. Knee Surg Sports Traumatol Arthrosc. (2025). doi: 10.1002/ksa.12605, PMID: (online ahead of print). 39901823 PMC12205424

[19] Della Rocca G. Initial psychometric testing and validation of the Italian version of the canine brief pain inventory in dogs with pain related to osteoarthritis. Front Vet Sci. (2021) 8:736458. doi: 10.3389/fvets.2021.736458, PMID: 34604372 PMC8484962

[20] Essner A Zetterberg L Hellström K Gustås P Högberg H Sjöström R. Psychometric evaluation of the canine brief pain inventory in a Swedish sample of dogs with pain related to osteoarthritis. Acta Vet Scand. (2017) 59:44. doi: 10.1186/s13028-017-0311-2, PMID: 28668080 PMC5493851

[21] Mazzuca SA. Imaging and osteoarthritis: what is the predictive value? Curr Rheumatol Rep. (2003) 5:27–32. doi: 10.1007/s11926-003-0080-y, PMID: 12590882

[22] Abadie E Ethgen D Avouac B Bouvenot G Branco J Bruyere O. Recommendations for the use of new methods to assess the efficacy of disease-modifying drugs in the treatment of osteoarthritis. Osteoarthr Cartil. (2004) 12:263–8. doi: 10.1016/j.joca.2004.01.006, PMID: 15023377

[23] Roux CH Foltz V Maheu E Baron G Gandjbakhch F Lukas C. MRI and serum biomarkers correlate with radiographic features in painful hand osteoarthritis. Clin Exp Rheumatol. (2016) 34:991–8. PMID: 27749237

[24] Davey MS Davey MG Kenny P Gheiti AJC. The use of radiomic analysis of magnetic resonance imaging findings in predicting features of early osteoarthritis of the knee-a systematic review and meta-analysis. Ir J Med Sci. (2024) 193:2525–30. doi: 10.1007/s11845-024-03714-5, PMID: 38822185 PMC11450002

[25] Raynauld JP Martel-Pelletier J Berthiaume MJ Beaudoin G Choquette D Haraoui B. Long term evaluation of disease progression through the quantitative magnetic resonance imaging of symptomatic knee osteoarthritis patients: correlation with clinical symptoms and radiographic changes. Arthritis Res Ther. (2006) 8:R21. doi: 10.1186/ar1875, PMID: 16507119 PMC1526551

[26] Wang XZ Zheng YX Cao YL Gu XF Wei SP Gao NY. Study on the diagnostic value of whole-organ magnetic resonance imaging score (WORMS) in knee osteoarthritis. Zhongguo Gu Shang. (2012) 25:364–8. PMID: 22870677

[27] Kauppinen S Fercher D Barreto G Karjalainen VP Virtanen V Baixauli-Marin L. Assessment of whole cartilage surface damage in an osteoarthritis rat model: the cartilage roughness score (CRS) utilizing microcomputed tomography. Osteoarthr Cartil. (2025) 33:134–45. doi: 10.1016/j.joca.2024.09.008, PMID: 39357597

[28] Atar MO Özçakar L Gençtürk Z Aytür Y. Serum endothelin-1 levels, radiographic and ultrasonographic evaluations, and clinical parameters in patients with knee and/or hand osteoarthritis. J Back Musculoskelet Rehabil. (2019) 32:549–54. doi: 10.3233/BMR-181326, PMID: 30530965

[29] Bassiouni HM el-Deeb M Kenawy N Abdul-Azim E Khairy M. Phonoarthrography, musculoskeletal ultrasonography, and biochemical biomarkers for the evaluation of knee cartilage in osteoarthritis. Mod Rheumatol. (2011) 21:500–8. doi: 10.3109/s10165-011-0441-8, PMID: 21442436

[30] Meehan R Wilson C Hoffman E Altimier L Kaessner M Regan EA. Ultrasound measurement of knee synovial fluid during external pneumatic compression. J Orthop Res. (2019) 37:601–8. doi: 10.1002/jor.24216, PMID: 30644131

[31] Kim HR Lee JH Kim KW Kim BM Lee SH. The relationship between synovial fluid VEGF and serum leptin with ultrasonographic findings in knee osteoarthritis. Int J Rheum Dis. (2016) 19:233–40. doi: 10.1111/1756-185X.12486, PMID: 25529922

[32] Monibi F Roller BL Stoker A Garner B Bal S Cook JL. Identification of synovial fluid biomarkers for knee osteoarthritis and correlation with radiographic assessment. J Knee Surg. (2016) 29:242–7. doi: 10.1055/s-0035-1549022, PMID: 25927354

[33] Izaguirre A González-Gutiérrez G Galindo-López SE Arenas-Serna G Rodríguez A Flores-Torrero E. Evaluation of biomarkers of joint damage in patients subjected to arthroscopy. Int Orthop. (2021) 45:1413–20. doi: 10.1007/s00264-020-04829-x, PMID: 33005990

[34] Bihlet AR Byrjalsen I Bay-Jensen AC Andersen JR Christiansen C Riis BJ. Associations between biomarkers of bone and cartilage turnover, gender, pain categories and radiographic severity in knee osteoarthritis. Arthritis Res Ther. (2019) 21:203. doi: 10.1186/s13075-019-1987-7, PMID: 31481084 PMC6724319

[35] Alilovic I Rukavina D Ajanovic A Eterovic T Milosevic H Ohran H. Breed-specific evaluation of serum biochemical markers in canine hip dysplasia observed in a Tornjak dog population. Am J Vet Res. (2023) 84:1–8. doi: 10.2460/ajvr.23.07.0170, PMID: 37699541

[36] Ilia I Ciordas PD Nitusca D Anton A Marian C. Analysis of the level of adiponectin and selected cytokines in patients with knee osteoarthritis. Medicina (Kaunas). (2024) 60:571. doi: 10.3390/medicina60040571 38674217 PMC11052232

[37] Song YZ Guan J Wang HJ Ma W Li F Xu F. Possible involvement of serum and synovial fluid Resistin in knee osteoarthritis: cartilage damage, clinical, and radiological links. J Clin Lab Anal. (2016) 30:437–43. doi: 10.1002/jcla.21876, PMID: 26494484 PMC6807051

[38] Garnero P Charni N Juillet F Conrozier T Vignon E. Increased urinary type II collagen helical and C telopeptide levels are independently associated with a rapidly destructive hip osteoarthritis. Ann Rheum Dis. (2006) 65:1639–44. doi: 10.1136/ard.2006.052621, PMID: 16569684 PMC1798449

[39] Pavelka K Forejtová Š Olejárová M Gatterová J Šenolt L Špaček P. Hyaluronic acid levels may have predictive value for the progression of knee osteoarthritis. Osteoarthr Cartil. (2004) 12:277–83. doi: 10.1016/j.joca.2004.01.001, PMID: 15023379

[40] Henrotin Y Sanchez C Balligand M. Pharmaceutical and nutraceutical management of canine osteoarthritis: present and future perspectives. Vet J. (2005) 170:113–23. doi: 10.1016/j.tvjl.2004.08.014, PMID: 15993795

[41] Song X Liu Y Chen S Zhang L Zhang H Shen X. Knee osteoarthritis: a review of animal models and intervention of traditional Chinese medicine. Animal Model Exp Med. (2024) 7:114–26. doi: 10.1002/ame2.12389, PMID: 38409942 PMC11079151

[42] Papich MG. An update on nonsteroidal anti-inflammatory drugs (NSAIDs) in small animals. Vet Clin North Am Small Anim Pract. (2008) 38:1243–66. doi: 10.1016/j.cvsm.2008.09.002, PMID: 18954683

[43] White C Morrow L. Efficacy of meloxicam compared with carprofen for treating canine osteoarthritis. Vet Rec. (2020) 186:94. doi: 10.1136/vr.m50, PMID: 31974182

[44] Kongara K Chambers JP. Robenacoxib in the treatment of pain in cats and dogs: safety, efficacy, and place in therapy. Vet Med (Auckl). (2018) 9:53–61. doi: 10.2147/VMRR.S170893 30148083 PMC6101027

[45] Corsato Alvarenga I Panickar KS Hess H McGrath S. Scientific validation of Cannabidiol for Management of dog and cat Diseases. Annu Rev Anim Biosci. (2023) 11:227–46. doi: 10.1146/annurev-animal-081122-070236 36790884

[46] Bhathal A Spryszak M Louizos C Frankel G. Glucosamine and chondroitin use in canines for osteoarthritis: a review. Open Vet J. (2017) 7:36–49. doi: 10.4314/ovj.v7i1.6, PMID: 28331832 PMC5356289

[47] Gross DM. Introduction to therapeutic lasers in a rehabilitation setting. Top Companion Anim Med. (2014) 29:49–53. doi: 10.1053/j.tcam.2014.09.004, PMID: 25454376

[48] Barbeau-Gregoire M. A 2022 systematic review and Meta-analysis of enriched therapeutic diets and nutraceuticals in canine and feline osteoarthritis. Int J Mol Sci. (2022) 23:10384. doi: 10.3390/ijms231810384, PMID: 36142319 PMC9499673

[49] Morita M Yamada K Date H Hayakawa K Sakurai H Yamada H. Efficacy of chondroitin sulfate for painful knee osteoarthritis: a one-year, randomized, double-blind, multicenter clinical study in Japan. Biol Pharm Bull. (2018) 41:163–71. doi: 10.1248/bpb.b17-00556, PMID: 29176264

[50] Bostrom A. Systematic review of complementary and alternative veterinary medicine in sport and companion animals: extracorporeal shockwave therapy. Animals (Basel). (2022) 12:3124. doi: 10.3390/ani12223124, PMID: 36428352 PMC9686741

[51] Bostrom A. Systematic review of complementary and alternative veterinary medicine in sport and companion animals: therapeutic ultrasound. Animals (Basel). (2022) 12:3144. doi: 10.3390/ani12223144, PMID: 36428372 PMC9686477

[52] Vargel I Tuncel A Baysal N Hartuç-Çevik İ Korkusuz F. Autologous adipose-derived tissue stromal vascular fraction (AD-tSVF) for knee osteoarthritis. Int J Mol Sci. (2022) 23:13517. doi: 10.3390/ijms232113517, PMID: 36362308 PMC9658499

[53] Lana J Lana AVSD da Fonseca LF Coelho MA Marques GG Mosaner T. Stromal vascular fraction for knee osteoarthritis - an update. J Stem Cells Regen Med. (2022) 18:11–20. doi: 10.46582/jsrm.1801003, PMID: 36003656 PMC9379357

[54] Upchurch DA Renberg WC Roush JK Milliken GA Weiss ML. Effects of administration of adipose-derived stromal vascular fraction and platelet-rich plasma to dogs with osteoarthritis of the hip joints. Am J Vet Res. (2016) 77:940–51. doi: 10.2460/ajvr.77.9.940, PMID: 27580105

[55] Ossendorff R Menon A Schildberg FA Randelli PS Scheidt S Burger C. A worldwide analysis of adipose-derived stem cells and stromal vascular fraction in orthopedics: current evidence and applications. J Clin Med. (2023) 12:4719. doi: 10.3390/jcm12144719, PMID: 37510834 PMC10380598

[56] Schroers M Bruns Y Waselau AC Steigmeier-Raith S Meyer-Lindenberg A. Autologous point-of-care stromal vascular fraction transplantation in dogs with advanced osteoarthritis of the knee and hip joints. Aust Vet J. (2024) 102:41–6. doi: 10.1111/avj.13303, PMID: 38044819

[57] Boada-Pladellorens A Avellanet M Pages-Bolibar E Veiga A. Stromal vascular fraction therapy for knee osteoarthritis: a systematic review. Ther Adv Musculoskelet Dis. (2022) 14:1759720X221117879. doi: 10.1177/1759720X221117879, PMID: 35991523 PMC9386815

[58] Kemilew J. The use of allogenic stromal vascular fraction (SVF) cells in degenerative joint disease of the spine in dogs. In Vivo. (2019) 33:1109–17. doi: 10.21873/invivo.11580, PMID: 31280199 PMC6689365

[59] Bruns Y Schroers M Steigmeier-Raith S Waselau AC Reese S Meyer-Lindenberg A. Efficacy of a single injection of stromal vascular fraction in dogs with elbow osteoarthritis: a clinical prospective study. Animals (Basel). (2024) 14:2803. doi: 10.3390/ani14192803 39409752 PMC11475770

[60] Prislin M. An outstanding role of adipose tissue in canine stem cell therapy. Animals (Basel). (2022) 12:1088. doi: 10.3390/ani12091088 35565514 PMC9099541

[61] Fotouhi A Maleki A Dolati S Aghebati-Maleki A Aghebati-Maleki L. Platelet rich plasma, stromal vascular fraction and autologous conditioned serum in treatment of knee osteoarthritis. Biomed Pharmacother. (2018) 104:652–60. doi: 10.1016/j.biopha.2018.05.019, PMID: 29803179

[62] Bora P Majumdar AS. Adipose tissue-derived stromal vascular fraction in regenerative medicine: a brief review on biology and translation. Stem Cell Res Ther. (2017) 8:145. doi: 10.1186/s13287-017-0598-y, PMID: 28619097 PMC5472998

[63] Tang Q Zhao XS Guo A Cui RT Song HL Qi ZY. Therapeutic applications of adipose-derived stromal vascular fractions in osteoarthritis. World J Stem Cells. (2022) 14:744–55. doi: 10.4252/wjsc.v14.i10.744, PMID: 36337155 PMC9630988

[64] Guo J Nguyen A Banyard DA Fadavi D Toranto JD Wirth GA. Stromal vascular fraction: a regenerative reality? Part 2: mechanisms of regenerative action. J Plast Reconstr Aesthet Surg. (2016) 69:180–8. doi: 10.1016/j.bjps.2015.10.014, PMID: 26546112

[65] Andia I Maffulli N Burgos-Alonso N. Stromal vascular fraction technologies and clinical applications. Expert Opin Biol Ther. (2019) 19:1289–305. doi: 10.1080/14712598.2019.1671970, PMID: 31544555

[66] Gareev I Beylerli O Ilyasova T Ahmad A Shi H Chekhonin V. Therapeutic application of adipose-derived stromal vascular fraction in myocardial infarction. iScience. (2024) 27:109791. doi: 10.1016/j.isci.2024.109791, PMID: 38736548 PMC11088339

[67] Uguten M van der Sluis N Vriend L Coert JH Harmsen MC van der Lei B. Comparing mechanical and enzymatic isolation procedures to isolate adipose-derived stromal vascular fraction: a systematic review. Wound Repair Regen. (2024) 32:1008–21. doi: 10.1111/wrr.13228, PMID: 39444305 PMC11584359

[68] Lockhart RA Aronowitz JA Dos-Anjos Vilaboa S. Use of freshly isolated human adipose stromal cells for clinical applications. Aesthet Surg J. (2017) 37:S4–8. doi: 10.1093/asj/sjw270, PMID: 29025212

[69] Pak J Lee JH Pak N Pak Y Park KS Jeon JH. Cartilage regeneration in humans with adipose tissue-derived stem cells and adipose stromal vascular fraction cells: updated status. Int J Mol Sci. (2018) 19:2146. doi: 10.3390/ijms19072146, PMID: 30041472 PMC6073159

[70] Zhang Y Chen X Tong Y Luo J Bi Q. Development and Prospect of intra-articular injection in the treatment of osteoarthritis: a review. J Pain Res. (2020) 13:1941–55. doi: 10.2147/JPR.S260878, PMID: 32801850 PMC7414982

[71] Ferreira MY Carvalho Junior JDC Ferreira LM. Evaluating the quality of studies reporting on clinical applications of stromal vascular fraction: a systematic review and proposed reporting guidelines (CLINIC-STRA-SVF). Regen Ther. (2023) 24:332–42. doi: 10.1016/j.reth.2023.08.003, PMID: 37662694 PMC10474569

[72] Goncharov EN. Analyzing the clinical potential of stromal vascular fraction: a comprehensive literature review. Medicina (Kaunas). (2024) 60:221. doi: 10.3390/medicina60020221, PMID: 38399509 PMC10890435

[73] Dubey NK Mishra VK Dubey R Deng YH Tsai FC Deng WP. Revisiting the advances in isolation, characterization and Secretome of adipose-derived stromal/stem cells. Int J Mol Sci. (2018) 19:2200. doi: 10.3390/ijms19082200, PMID: 30060511 PMC6121360

[74] Brown JC Shang H Li Y Yang N Patel N Katz AJ. Isolation of adipose-derived stromal vascular fraction cells using a novel point-of-care device: cell characterization and review of the literature. Tissue Eng Part C Methods. (2017) 23:125–35. doi: 10.1089/ten.tec.2016.0377, PMID: 28177263

[75] Lee SJ Lee CR Kim KJ Ryu YH Kim E Han YN. Optimal condition of isolation from an adipose tissue-derived stromal vascular fraction for the development of automated systems. Tissue Eng Regen Med. (2020) 17:203–8. doi: 10.1007/s13770-019-00238-3, PMID: 31997256 PMC7105537

[76] Harasymiak-Krzyzanowska I. Adipose tissue-derived stem cells show considerable promise for regenerative medicine applications. Cell Mol Biol Lett. (2013) 18:479–93. doi: 10.2478/s11658-013-0101-4, PMID: 23949841 PMC6275722

[77] Deptula M. Adipose-derived stromal cells for nonhealing wounds: emerging opportunities and challenges. Med Res Rev. (2021) 41:2130–71. doi: 10.1002/med.21789, PMID: 33522005 PMC8247932

[78] Monivas E. Comparison between manual and automated methods for the isolation of mononuclear cells and mesenchymal stem cells using ficoll: efficacy and reproducibility. Front Cell Dev Biol. (2025) 13:1556697. doi: 10.3389/fcell.2025.1556697, PMID: 40191228 PMC11969043

[79] Song N Scholtemeijer M Shah K. Mesenchymal stem cell immunomodulation: mechanisms and therapeutic potential. Trends Pharmacol Sci. (2020) 41:653–64. doi: 10.1016/j.tips.2020.06.009, PMID: 32709406 PMC7751844

[80] Fan XL Zhang Y Li X Fu QL. Mechanisms underlying the protective effects of mesenchymal stem cell-based therapy. Cell Mol Life Sci. (2020) 77:2771–94. doi: 10.1007/s00018-020-03454-6, PMID: 31965214 PMC7223321

[81] Anjiki K Matsumoto T Kuroda Y Fujita M Hayashi S Nakano N. Heterogeneous cells as well as adipose-derived stromal cells in stromal vascular fraction contribute to enhance anabolic and inhibit catabolic factors in osteoarthritis. Stem Cell Rev Rep. (2023) 19:2407–19. doi: 10.1007/s12015-023-10589-z, PMID: 37477775

[82] Bergstrom A. Long-term effect of intra-articular adipose-derived stromal vascular fraction and platelet-rich plasma in dogs with elbow joint disease-a pilot study. Vet Sci. (2024) 11:296. doi: 10.3390/vetsci11070296, PMID: 39057980 PMC11281594

[83] Franklin SP Stoker AM Bozynski CC Kuroki K Clarke KM Johnson JK. Comparison of platelet-rich plasma, stromal vascular fraction (SVF), or SVF with an injectable PLGA nanofiber scaffold for the treatment of Osteochondral injury in dogs. J Knee Surg. (2018) 31:686–97. doi: 10.1055/s-0037-1606575, PMID: 28915522

[84] Marx C Silveira MD Beyer Nardi N. Adipose-derived stem cells in veterinary medicine: characterization and therapeutic applications. Stem Cells Dev. (2015) 24:803–13. doi: 10.1089/scd.2014.0407, PMID: 25556829

[85] Marx C. Acupoint injection of autologous stromal vascular fraction and allogeneic adipose-derived stem cells to treat hip dysplasia in dogs. Stem Cells Int. (2014) 2014:391274. doi: 10.1155/2014/391274 25180040 PMC4144304

[86] Meena A D’Ambrosi R Farinelli L Attri M Mabrouk A Nakamura N. Should I add orthobiologics to my knee osteotomy practice? A systematic review. J ISAKOS. (2024) 9:100282. doi: 10.1016/j.jisako.2024.06.001, PMID: 38851324

[87] Mehranfar S Abdi Rad I Mostafavi E Akbarzadeh A. The use of stromal vascular fraction (SVF), platelet-rich plasma (PRP) and stem cells in the treatment of osteoarthritis: an overview of clinical trials. Artif Cells Nanomed Biotechnol. (2019) 47:882–90. doi: 10.1080/21691401.2019.1576710, PMID: 30887856

[88] Bertheuil N Chaput B Ménard C Varin A Laloze J Watier E. Adipose mesenchymal stromal cells: definition, immunomodulatory properties, mechanical isolation and interest for plastic surgery. Ann Chir Plast Esthet. (2019) 64:1–10. doi: 10.1016/j.anplas.2018.07.005, PMID: 30126741

[89] De Francesco F. Human adipose stem cells: from bench to bedside. Tissue Eng Part B Rev. (2015) 21:572–84. doi: 10.1089/ten.teb.2014.0608, PMID: 25953464

[90] Klar AS Zimoch J Biedermann T. Skin tissue engineering: application of adipose-derived stem cells. Biomed Res Int. (2017) 2017:9747010. doi: 10.1155/2017/9747010 28337463 PMC5350314

[91] Ong WK Sugii S. Adipose-derived stem cells: fatty potentials for therapy. Int J Biochem Cell Biol. (2013) 45:1083–6. doi: 10.1016/j.biocel.2013.02.013, PMID: 23458962

[92] Pak J Lee JH Park KS Park M Kang LW Lee SH. Current use of autologous adipose tissue-derived stromal vascular fraction cells for orthopedic applications. J Biomed Sci. (2017) 24:9. doi: 10.1186/s12929-017-0318-z, PMID: 28143470 PMC5282826

[93] Ong WK Chakraborty S Sugii S. Adipose tissue: understanding the heterogeneity of stem cells for regenerative medicine. Biomol Ther. (2021) 11:918. doi: 10.3390/biom11070918, PMID: 34206204 PMC8301750

[94] Malekzadeh H Tirmizi Z Arellano JA Egro FM Ejaz A. Application of adipose-tissue derived products for burn wound healing. Pharmaceuticals (Basel). (2023) 16:1302. doi: 10.3390/ph16091302, PMID: 37765109 PMC10534650

[95] Jeyaraman N Shrivastava S Ravi VR Nallakumarasamy A Pundkar A Jeyaraman M. Understanding and controlling the variables for stromal vascular fraction therapy. World J Stem Cells. (2024) 16:784–98. doi: 10.4252/wjsc.v16.i8.784, PMID: 39219728 PMC11362852

[96] Zhao Y Xie L. An update on mesenchymal stem cell-centered therapies in temporomandibular joint osteoarthritis. Stem Cells Int. (2021) 2021:6619527. doi: 10.1155/2021/6619527 33868408 PMC8035039

[97] Kalluri R LeBleu VS. The biology, function, and biomedical applications of exosomes. Science. (2020) 367:eaau6977. doi: 10.1126/science.aau6977, PMID: 32029601 PMC7717626

[98] Suh A Pham A Cress MJ Pincelli T TerKonda SP Bruce AJ. Adipose-derived cellular and cell-derived regenerative therapies in dermatology and aesthetic rejuvenation. Ageing Res Rev. (2019) 54:100933. doi: 10.1016/j.arr.2019.100933, PMID: 31247326

[99] Jafarzadeh A Mohammad AP Goodarzi A. A systematic review of case series and clinical trials investigating regenerative medicine for the treatment of vitiligo. J Cosmet Dermatol. (2024) 24:e16660. doi: 10.1111/jocd.16660, PMID: 39509558 PMC11847760

[100] Jiang S Quan Y Wang J Cai J Lu F. Fat grafting for facial rejuvenation using stromal vascular fraction gel injection. Clin Plast Surg. (2020) 47:73–9. doi: 10.1016/j.cps.2019.09.001 31739900

[101] Raposo G Stoorvogel W. Extracellular vesicles: exosomes, microvesicles, and friends. J Cell Biol. (2013) 200:373–83. doi: 10.1083/jcb.201211138, PMID: 23420871 PMC3575529

[102] Harrell CR Jovicic N Djonov V Arsenijevic N Volarevic V. Mesenchymal stem cell-derived exosomes and other extracellular vesicles as new remedies in the therapy of inflammatory diseases. Cells. (2019) 8:1605. doi: 10.3390/cells8121605, PMID: 31835680 PMC6952783

[103] Chung YH Ho YP Farn SS Tsai WC Li ZX Lin TY. In vivo SPECT imaging of Tc-99m radiolabeled exosomes from human umbilical-cord derived mesenchymal stem cells in small animals. Biom J. (2024) 47:100721. doi: 10.1016/j.bj.2024.100721, PMID: 38636899 PMC11401219

[104] Tang N. Exosomes in multiple sclerosis and Alzheimer's disease - adversary and ally. Biom J. (2024) 47:100665. doi: 10.1016/j.bj.2023.100665, PMID: 37778696 PMC11401191

[105] Wang AYL. Human induced pluripotent stem cell-derived exosomes as a new therapeutic strategy for various diseases. Int J Mol Sci. (2021) 22:1769. doi: 10.3390/ijms22041769, PMID: 33578948 PMC7916646

[106] Hsu HH Wang AYL Loh CYY Pai AA Kao HK. Therapeutic potential of exosomes derived from diabetic adipose stem cells in cutaneous wound healing of db/db mice. Pharmaceutics. (2022) 14:1206. doi: 10.3390/pharmaceutics14061206, PMID: 35745779 PMC9227821

[107] Saba E Sandhu MA Pelagalli A. Canine mesenchymal stromal cell exosomes: state-of-the-art characterization, functional analysis and applications in various diseases. Vet Sci. (2024) 11:187. doi: 10.3390/vetsci11050187, PMID: 38787159 PMC11126113

[108] Bharti D Ajith Y Sharun K Banu SA Kumar A Bhardwaj A. Therapeutic applications of canine platelets and their derivatives: a narrative review. Top Companion Anim Med. (2024) 58:100840. doi: 10.1016/j.tcam.2023.100840, PMID: 37979613

[109] Anil U Markus DH Hurley ET Manjunath AK Alaia MJ Campbell KA. The efficacy of intra-articular injections in the treatment of knee osteoarthritis: a network meta-analysis of randomized controlled trials. Knee. (2021) 32:173–82. doi: 10.1016/j.knee.2021.08.008, PMID: 34500430

[110] Rodriguez-Merchan EC. Autologous and allogenic utilization of stromal vascular fraction and Decellularized extracellular matrices in orthopedic surgery: a scoping review. Arch Bone Jt Surg. (2022) 10:827–32. doi: 10.22038/ABJS.2022.59635.2943, PMID: 36452418 PMC9702025

[111] Bergh MS Budsberg SC. The coxib NSAIDs: potential clinical and pharmacologic importance in veterinary medicine. J Vet Intern Med. (2005) 19:633–43. doi: 10.1111/j.1939-1676.2005.tb02741.x, PMID: 16231707

[112] Lees P. Pharmacodynamics and pharmacokinetics of nonsteroidal anti-inflammatory drugs in species of veterinary interest. J Vet Pharmacol Ther. (2004) 27:479–90. doi: 10.1111/j.1365-2885.2004.00617.x, PMID: 15601442

[113] Ricketts AP Lundy KM Seibel SB. Evaluation of selective inhibition of canine cyclooxygenase 1 and 2 by carprofen and other nonsteroidal anti-inflammatory drugs. Am J Vet Res. (1998) 59:1441–6. doi: 10.2460/ajvr.1998.59.11.1441, PMID: 9829404

[114] Lascelles BD McFarland JM Swann H. Guidelines for safe and effective use of NSAIDs in dogs. Vet Ther. (2005) 6:237–51. PMID: 16299670

[115] Punke JP Speas AL Reynolds LR Budsberg SC. Effects of firocoxib, meloxicam, and tepoxalin on prostanoid and leukotriene production by duodenal mucosa and other tissues of osteoarthritic dogs. Am J Vet Res. (2008) 69:1203–9. doi: 10.2460/ajvr.69.9.1203, PMID: 18764695

[116] Block JA Oegema TR Sandy JD Plaas A. The effects of oral glucosamine on joint health: is a change in research approach needed? Osteoarthr Cartil. (2010) 18:5–11. doi: 10.1016/j.joca.2009.07.005, PMID: 19733270

[117] Bassleer C Rovati L Franchimont P. Stimulation of proteoglycan production by glucosamine sulfate in chondrocytes isolated from human osteoarthritic articular cartilage in vitro. Osteoarthr Cartil. (1998) 6:427–34. doi: 10.1053/joca.1998.0146, PMID: 10343776

[118] Piperno M Reboul P Hellio le Graverand MP Peschard MJ Annefeld M Richard M. Glucosamine sulfate modulates dysregulated activities of human osteoarthritic chondrocytes in vitro. Osteoarthr Cartil. (2000) 8:207–12. doi: 10.1053/joca.1999.0291, PMID: 10806048

[119] Largo R Alvarez-Soria MA Díez-Ortego I Calvo E Sánchez-Pernaute O Egido J. Glucosamine inhibits IL-1beta-induced NFkappaB activation in human osteoarthritic chondrocytes. Osteoarthr Cartil. (2003) 11:290–8. doi: 10.1016/S1063-4584(03)00028-1, PMID: 12681956

[120] Reginster JY Deroisy R Rovati LC Lee RL Lejeune E Bruyere O. Long-term effects of glucosamine sulphate on osteoarthritis progression: a randomised, placebo-controlled clinical trial. Lancet. (2001) 357:251–6. doi: 10.1016/S0140-6736(00)03610-2, PMID: 11214126

[121] Bierer TL Bui LM. Improvement of arthritic signs in dogs fed green-lipped mussel (Perna canaliculus). J Nutr. (2002) 132:1634S–6S. doi: 10.1093/jn/132.6.1634S, PMID: 12042477

[122] Henrotin YE Sanchez C Deberg MA Piccardi N Guillou GB Msika P. Avocado/soybean unsaponifiables increase aggrecan synthesis and reduce catabolic and proinflammatory mediator production by human osteoarthritic chondrocytes. J Rheumatol. (2003) 30:1825–34. PMID: 12913942

[123] Pelletier JP Lajeunesse D Jovanovic DV Lascau-Coman V Jolicoeur FC Hilal G. Carprofen simultaneously reduces progression of morphological changes in cartilage and subchondral bone in experimental dog osteoarthritis. J Rheumatol. (2000) 27:2893–902. 11128682

[124] Chamberlain GA Colborne GR. A review of the cellular and molecular effects of extracorporeal shockwave therapy. Vet Comp Orthop Traumatol. (2016) 29:99–107. doi: 10.3415/VCOT-15-04-0057, PMID: 26846274

[125] Robertson VJ Baker KG. A review of therapeutic ultrasound: effectiveness studies. Phys Ther. (2001) 81:1339–50. doi: 10.1093/ptj/81.7.1339 11444997

[126] Frisbie DD Kawcak CE McIlwraith CW. Evaluation of the effect of extracorporeal shock wave treatment on experimentally induced osteoarthritis in middle carpal joints of horses. Am J Vet Res. (2009) 70:449–54. doi: 10.2460/ajvr.70.4.449, PMID: 19335099

[127] Dahlberg JA McClure SR Evans RB Reinertson EL. Force platform evaluation of lameness severity following extracorporeal shock wave therapy in horses with unilateral forelimb lameness. J Am Vet Med Assoc. (2006) 229:100–3. doi: 10.2460/javma.229.1.100, PMID: 16817722

[128] Mueller M Bockstahler B Skalicky M Mlacnik E Lorinson D. Effects of radial shockwave therapy on the limb function of dogs with hip osteoarthritis. Vet Rec. (2007) 160:762–5. doi: 10.1136/vr.160.22.762, PMID: 17545646

[129] Brown KE. Investigation of the immediate analgesic effects of extracorporeal shock wave therapy for treatment of navicular disease in horses. Vet Surg. (2005) 34:554–8. doi: 10.1111/j.1532-950X.2005.00087.x, PMID: 16343141

[130] Byron C Stewart A Benson B Tennent-Brown B Foreman J. Effects of radial extracorporeal shock wave therapy on radiographic and scintigraphic outcomes in horses with palmar heel pain. Vet Comp Orthop Traumatol. (2009) 22:113–8. doi: 10.3415/VCOT-08-04-0037, PMID: 19290391

[131] Hendawy H Uemura A Ma D Namiki R Samir H Ahmed MF. Tissue harvesting site effect on the canine adipose stromal vascular fraction quantity and quality. Animals (Basel). (2021) 11:460. doi: 10.3390/ani11020460, PMID: 33572472 PMC7916364

[132] Wu LW Wang YL Christensen JM Khalifian S Schneeberger S Raimondi G. Donor age negatively affects the immunoregulatory properties of both adipose and bone marrow derived mesenchymal stem cells. Transpl Immunol. (2014) 30:122–7. doi: 10.1016/j.trim.2014.03.001, PMID: 24632513

[133] Lee HY Bin SI Kim JM Lee BS Kim SM Lee SJ. Nonextruded grafts result in better cartilage quality after lateral meniscal allograft transplantation: quantitative 3-T MRI T2 mapping. Am J Sports Med. (2023) 51:404–12. doi: 10.1177/03635465221143373, PMID: 36607167

[134] Jerban S Kasibhatla A Ma Y Wu M Chen Y Guo T. Detecting articular cartilage and Meniscus deformation effects using magnetization transfer ultrashort Echo time (MT-UTE) modeling during mechanical load application: ex vivo feasibility study. Cartilage. (2021) 13:665S–73S. doi: 10.1177/1947603520976771, PMID: 33289401 PMC8808840

[135] Zhang K Yu J Li J Fu W. The combined intraosseous Administration of Orthobiologics Outperformed Isolated Intra-articular Injections in alleviating pain and cartilage degeneration in a rat model of MIA-induced knee osteoarthritis. Am J Sports Med. (2024) 52:140–54. doi: 10.1177/03635465231212668, PMID: 38164685

[136] Tang X Guo D Liu A Wu D Liu J Xu N. Fully automatic knee joint segmentation and quantitative analysis for osteoarthritis from magnetic resonance (MR) images using a deep learning model. Med Sci Monit. (2022) 28:e936733. doi: 10.12659/MSM.936733, PMID: 35698440 PMC9206408

